# PedVacc 002: A phase I/II randomized clinical trial of MVA.HIVA vaccine administered to infants born to human immunodeficiency virus type 1-positive mothers in Nairobi

**DOI:** 10.1016/j.vaccine.2014.08.034

**Published:** 2014-10-07

**Authors:** Irene N. Njuguna, Gwen Ambler, Marie Reilly, Beatrice Ondondo, Mercy Kanyugo, Barbara Lohman-Payne, Christine Gichuhi, Nicola Borthwick, Antony Black, Shams-Rony Mehedi, Jiyu Sun, Elizabeth Maleche-Obimbo, Bhavna Chohan, Grace C. John-Stewart, Walter Jaoko, Tomáš Hanke

**Affiliations:** a Department of Pediatrics and Child Health, University of Nairobi, PO Box 19676, 00202 Nairobi, Kenya; b Department of Global Health, University of Washington, Seattle, WA 98104, USA; c Department of Medical Epidemiology and Biostatistics, Karolinska Institute, SE-17177 Stockholm, Sweden; d The Jenner Institute, University of Oxford, Oxford OX3 7DQ, UK; e Department of Clinical Medicine and Therapeutics, University of Nairobi, PO Box 19676, 00202 Nairobi, Kenya; f Statistics and Data Management Department, Medical Research Council Unit, Fajara, The Gambia; g Departments of Pediatrics, Medicine, Epidemiology, and Global Health, University of Washington, Seattle, WA 98104, USA; h KAVI-Institute of Clinical Research, University of Nairobi, PO Box 19676, 00202 Nairobi, Kenya; i The Weatherall Institute of Molecular Medicine, University of Oxford, Oxford OX3 9DS, UK

**Keywords:** Modified vaccinia virus Ankara (MVA), Infant vaccine trial in Africa, Exposed-uninfected infants, HIV-1, Pediatric HIV-1 vaccines, KEPI vaccines, EPI vaccines, rMVA, recombinant modified vaccinia virus Ankara, HIV-1, human immunodeficiency virus type 1, HEU, HIV-1-exposed uninfected, ART, antiretroviral therapy, PMTCT, prevention of mother-to-child transmission of HIV-1, ZDV, zidovudine, TDF, tenofovir, 3TC, lamivudine, LPV/RTV, lopinavir/ritonavir, EFV, efavirenz, NVP, nevirapine, HBV, hepatitis B virus, OPV, oral polio vaccine, KEPI, Kenyan Expanded Program on Immunization, Dtx, diphtheria toxin, Ttx, tetanus toxin (Ttx), Hib, *Hemophilus influenzae* type b, KNH, Kenyatta National Hospital, IQR, interquartile range, WAZ, weight-for-age Z-score

## Abstract

**Background:**

A safe, effective vaccine for breastfeeding infants born to HIV-1-positive mothers could complement antiretroviral therapy (ART) for prevention of mother-to-child transmission of HIV-1. To date, only a few HIV-1 vaccine candidates have been tested in infants.

**Trial design:**

A phase I/II randomized controlled trial PedVacc 002 was conducted to determine the safety and immunogenicity of a single, low dose of MVA.HIVA vaccine delivered intramuscularly to healthy 20-week-old infants born to HIV-1-positive mothers in Nairobi, Kenya.

**Methods:**

Pregnant HIV-1-positive women in the 2nd/3rd trimester of gestation were enrolled, provided with ART and self-selected their infant-feeding modality. Infants received nevirapine and cotrimoxazole prophylaxis. At 20 weeks of age, eligible HIV-1-negative infants were randomized to vaccine versus no-treatment arms and followed to 48 weeks of age for assessments of vaccine safety, HIV-1-specific T-cell responses and antibodies to routine childhood vaccines.

**Results:**

Between February and November 2010, 182 mothers were screened, 104 were eligible and followed on ART during pregnancy/postpartum, of whom 73 had eligible infants at 20 weeks postpartum. Thirty-six infants were randomized to vaccine and 37 to no treatment. Eighty-four percent of infants breastfed, and retention at 48 weeks was 99%. Adverse events were rare and similar between the two arms. HIV-1-specific T-cell frequencies in interferon-γ ELISPOT assay were transiently higher in the MVA.HIVA arm (*p* = 0.002), but not above the threshold for a positive assay. Protective antibody levels were adequate and similar between arms for all routine childhood vaccines except HBV, where 71% of MVA.HIVA subjects compared to 92% of control subjects were protected (*p* = 0.05).

**Conclusions:**

This trial tested for the first time an MVA-vectored candidate HIV-1 vaccine in HIV-1-exposed infants in Africa, demonstrating trial feasibility and vaccine safety, low immunogenicity, and compatibility with routine childhood vaccinations. These results are reassuring for use of the MVA vector in more potent prime-boost regimens.

## Highlights

• MVA.HIVA vaccine was tested for the first time in HIV-1-exposed infants in Africa.
• PedVacc 002 had 99% retention of infants over 48 weeks of follow-up.
• MVA.HIVA was safe, but not sufficiently immunogenic.
• MVA.HVA did not interfere with routine childhood vaccines except for induction of HBV antibodies.
• MVA is well suited as a vaccine vector for infants under 1 year of age.

## 1 Introduction

In 2012, an estimated 260,000 children became infected with the human immunodeficiency virus type 1 (HIV-1) (www.unaids.org), the majority of whom acquired the virus from their mothers. The UNAIDS's ambitious *Global Plan towards The Elimination of New HIV-1 Infections among Children by 2015 and Keeping Their Mothers Alive* aims to decrease the number of new pediatric infections by 90% (www.unaids.org). Although the Global Plan is well underway, only an estimated 57% of HIV-1-positive pregnant women in low- and middle-income countries accessed appropriate prevention of mother-to-child HIV-1 transmission (PMTCT) antiretroviral regimens in 2012. Incomplete access to antiretroviral therapy (ART), ART side effects [1–8], non-adherence and/or HIV-1 drug resistance can lead to persistent risk of mother-to-child HIV-1 transmission despite expansion of PMTCT programs. Thus, effective HIV-1 vaccines to protect infants against breast milk HIV-1 transmission may complement and enhance current PMTCT strategies.

Vaccine prevention of breast milk HIV-1 transmission will require the priming vaccine to be administered within the first few days after birth, followed by boost(s) soon after. To date, there have been over 650 clinical studies assessing candidate HIV-1 vaccines in humans. However, fewer than 10 of these studies tested HIV-1 vaccine safety and immunogenicity in infants (www.clinicaltrials.gov), despite major differences in natural HIV-1 infection [9] and responsiveness to vaccinations [10,11] between adults and infants/young children. Infants have distinct characteristics that may influence their response to HIV-1 vaccines. Despite evidence of infants’ lower capacity for some immune responses, they have some potential advantages for generating responses. For example, infants have fewer competing memory T-cell clones that exist at the time of vaccination, making ‘space’ to establish new long-term cellular memory [12]. Thus, testing of candidate vaccines in pediatric populations is important for appropriate development of vaccines early in the pipeline [13].

One of the leading boosting vectors for genetic subunit vaccines is modified vaccinia virus Ankara (MVA), known for its excellent safety and immunogenicity record from human trials involving several thousands of individuals [14]. As an inroad for MVA-vectored HIV-1 vaccine use in infants, we tested a low dose of vaccine MVA.HIVA [15] in parallel in infants born to HIV-1-negative mothers (PedVacc 001 trial in The Gambia) and in infants born to HIV-1-positive mothers (PedVacc 002 trial in Kenya). MVA.HIVA delivered the first ever immunogen derived from an African clade A HIV-1 [16] to reach human evaluation in Africa.

When the PedVacc 001 and 002 trials were conceived in 2007, only three studies of active immunization in infants were published. These other studies evaluated 3 *Env*-derived subunit proteins and 3 canarypoxvirus (ALVAC)-vectored vaccines in Pediatric AIDS Clinical Trials Group protocols (PACTG) 320 [17–19] and 326 [20,21] and HIV Pediatric Trials Network (HPTN) protocol 027 [22]. The tested ALVAC and protein vaccines caused no increase in serious adverse events (SAE) and elicited promising immune responses similar to those observed in adults. We recently reported that the PedVacc 001 trial had excellent safety and marginal immunogenicity among 20-week-old Gambian infants born to HIV-1-negative mothers [23]. Here, we report on the administration of MVA.HIVA to infants born to HIV-1-positive mothers in Kenya (PedVacc 002) with the primary aim to assess its safety. This was the first time that a rMVA vaccine with an HIV-1-derived transgene was administered to infants born to HIV-1-positive mothers.

## 2 Materials and methods

### 2.1 Study design

The Pediatric Vaccine (PedVacc) 002 study was a single-site, phase I/II, open, randomized, controlled trial of candidate HIV-1 vaccine MVA.HIVA compared to no treatment. The primary outcome was MVA.HIVA vaccine safety.

### 2.2 Ethics and regulatory approvals

Approvals to conduct the study were granted by the Pharmacy and Poisons Board, Ministry of Medical Services, Kenya (ref. PPB/ECCT/08/25-2/10), Kenyatta National Hospital (KNH)/University of Nairobi Research Ethics Committee (ref. P266/10/2008), Nairobi University Institutional Biosafety Committee (ref. UON/CHS/PRINC/ADM1/SC6/IBC.CTTE/13), Oxford Tropical Research Ethics Committee (ref. OXTREC 52-08), University of Washington Institutional review Board (ref. HSD 35079), and the Stockholm Regional Ethics Committee (ref. 2009/1591-31/1). The study was conducted according to the principles of the Declaration of Helsinki (2008) and complied with the International Conference on Harmonization Good Clinical Practice guidelines.

### 2.3 Study population

The study was conducted at KNH in Nairobi, Kenya. HIV-1-positive pregnant women in their 2nd/3rd trimester were recruited from antenatal clinics at KNH and Nairobi City Council clinics. Women were eligible to participate if they were aged 18 years or above, had CD4^+^ cell count greater than 350 μl^−1^, WHO stage 1 or 2 disease, planned to deliver at KNH, and planned to remain in the Nairobi area for one year after delivery. Women in the study gave written informed consent and the infant's father, or other family member or significant person co-signed the consent form for participation. Mothers were provided with ART for PMTCT as per WHO Option B guidelines consisting of zidovudine (ZDV) or tenofovir (TDF), lamivudine (3TC), and lopinavir/ritonavir (LPV/RTV) or efavirenz (EFV) or nevirapine (NVP) during pregnancy, delivery and throughout breastfeeding. Women were counseled on feeding options and provided formula milk if they elected to use replacement feeding.

Within 3 days of birth, singleton infants were enrolled if they weighed at least 2.5 kg and did not have congenital defects or underlying disease that might compromise evaluation of response to the candidate vaccine. Infants received NVP prophylaxis for the first 6 weeks of life and cotrimoxazole prophylaxis from 6 weeks of age. Breastfeeding infants continued cotrimoxazole throughout the breastfeeding period while formula-fed infants stopped at 10 weeks if their 6-week HIV-1 test was negative. Infants received Kenyan Expanded Program on Immunization (KEPI) vaccinations, which included BCG and oral poliovirus vaccine (OPV) at birth, OPV and Pentavalent vaccine (diphtheria toxin [Dtx], tetanus toxin [Ttx], whole cell pertussis [Ptx], *Hemophilus influenzae* type b [Hib] and hepatitis B virus [HBV] surface antigen [HBsAg]) at 6, 10 and 14 weeks of age. Pneumoccocal conjugate vaccine 10, introduced in the course of the study was administered to infants at variable ages. During study visits, a standard questionnaire on infant health and immunization was completed. At 20 weeks, infants were randomized if they had received all scheduled KEPI vaccines, were HIV-1-uninfected, had weight-for-age *Z*-scores no more than 2 standard deviations below normal, had no acute or chronic disease, had no history of anaphylaxis reaction to prior vaccination, and baseline laboratory investigations were within normal ranges.

### 2.4 The study vaccine and its administration

MVA.HIVA is a recombinant non-replicating poxvirus, which carries the HIVA transgene inserted into the thymidine kinase locus of the parental MVA genome under the early/late P7.5 promoter [16]. MVA.HIVA was manufactured under current Good Manufacturing Practice conditions by IDT, Germany. It was provided in vials of 200 μl at 5 × 10^8^ plaque-forming units (PFU) ml^−1^ in 10 mM Tris–HCl buffer pH 7.7 and 0.9% NaCl, and stored at ≤−20 °C. On the day of administration, each vial was thawed at room temperature and given within 1 h of thawing. Infants randomized to vaccine group received a single intramuscular dose of 5 × 10^7^ pfu of MVA.HIVA, while the control group received no treatment. Vaccinated infants were observed in the clinic for 1 h post-vaccination and visited at home after 24 and 48 h to assess for adverse reactions.

### 2.5 Participant follow-up and safety monitoring

Randomization was generated at Karolinska Institute using a blocked design and participants were assigned using sealed envelopes. After randomization, medical history and examinations were conducted at 21, 28, 36 and 48 weeks of age. At 21 and 28 weeks, hematology and biochemistry tests were done as described below. Local, systemic and laboratory AEs, and relationship to MVA.HIVA were graded as per Clinical Protocol (Supplementary Information). Palpable lymph nodes, redness and induration were scored according to their diameters. Any Grade 3 or 4 laboratory AE was confirmed by re-test. An internal trial safety monitor reviewed Grade 3 and 4 events in real time and these were reported to the KNH Research Ethics committee. Study procedures were reviewed regularly by an external monitor. An external Data Monitoring and Ethics Committee reviewed safety data at 6-monthly intervals.

### 2.6 Blood sampling schedule and handling

Laboratory personnel were blind to group allocation. Five ml of blood (4 ml EDTA, 1 ml clotted) was collected at 19, 21, 28, 36 and 48 weeks of age. MVA.HIVA immunogenicity was tested at all 5 time points; hematology, biochemistry (including alanine transaminase [ALT] and creatinine tests), and CD4^+^ cell counts were conducted at 19, 21 and 28 weeks. KEPI vaccine antibody responses were determined at 19 and 21 weeks. HIV-1 testing was performed using HIV-1 DNA PCR at birth, 6, 10, 14 and 20 weeks; HIV-1 viral load at 19, 28, 36 and 48 weeks and HIV-ELISA at 48 weeks. Peripheral blood mononuclear cells (PBMC) were isolated and used for interferon (IFN)-γ ELISPOT assays or frozen [23].

### 2.7 IFN-γ ELISPOT assay

Fresh *ex vivo* and cultured IFN-γ ELISPOT assays were carried out as previously described [23]. An assay failed quality control if the mean background was >20 spot-forming units (SFU)/well (>100 SFU/10^6^ PBMC) or mean phytohemagglutinine response was <30 SFU/well (<150 SFU/10^6^ PBMC). A response was considered positive if the mean stimulated response was at least twice the mean background response and the net response (with background subtracted) was ≥50 SFU/10^6^ PBMC.

### 2.8 KEPI vaccine antibody responses

Microsphere-based multiplex assays were performed at the National Institute for Public Health and the Environment, Bilthoven, The Netherlands to quantify serum IgG antibodies against Ptx, Dtx, Ttx and Hib as described previously [24]. Anti-HBsAg antibody levels were measured using an anti-HBsAg enzyme immunoassay kit (ETI-AB-AUK-3, Diasorin, Italy). Type 1 poliovirus IgG levels were determined by a neutralization assay as described previously [25]. Infants with inadequate vaccine responses were offered revaccination.

### 2.9 Statistical analysis

Non-parametric tests were used to compare immune responses, hematology and biochemistry parameters. We reported local and systemic AEs occurring 8 weeks after vaccination. Infants could contribute to several AEs, and those with more than one report of the same event were assigned to the highest grade recorded for that condition if it was ongoing. If an event occurred in 2 or more distinct episodes, these were considered separate events. Two-tailed Mann–Whitney tests were used to compare the two trial randomization arms, and Wilcoxon matched-pairs tests assessed the changes in an infant's responses over time. The alpha level was set at <0.05 for statistical significance. Poisson models were used to examine replicate wells of the ELISPOT assays and extreme outliers that were identified (using a Bonferroni correction for multiple testing) were excluded prior to averaging. Data analysis was conducted with Stata version 12 (StataCorp, College Station, Texas).

## 3 Results

### 3.1 Trial participants

**Fig. 1:**
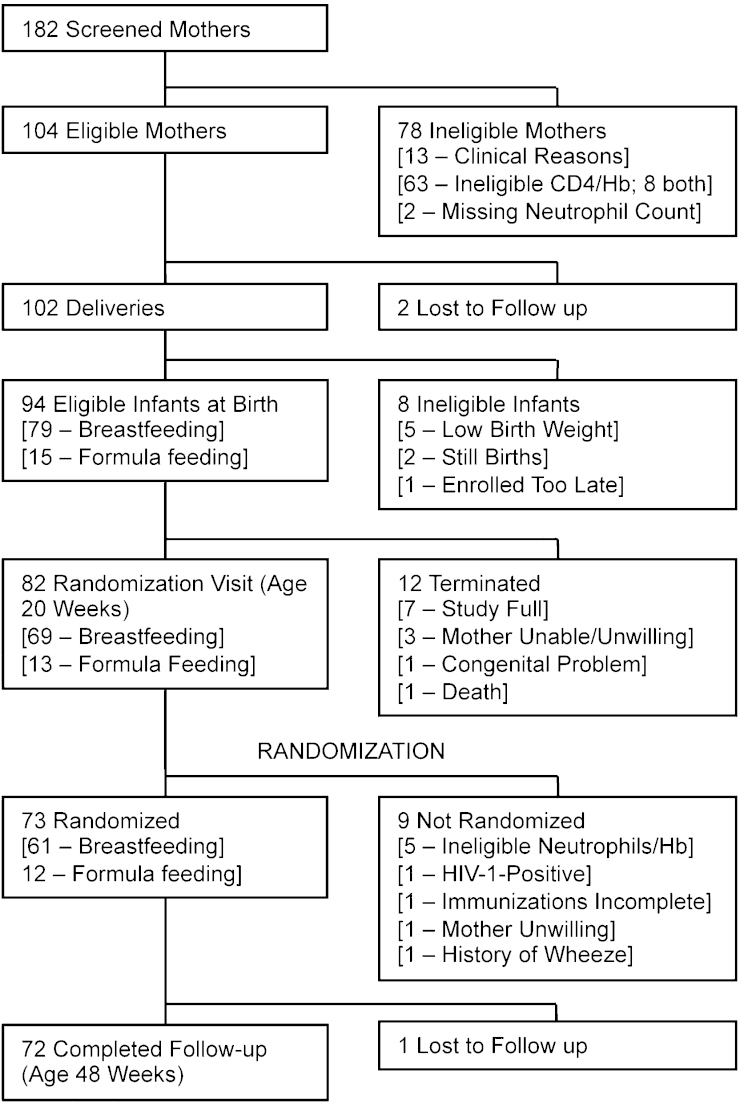
Trial profile. Diagram indicating the numbers of infants screened and followed up throughout the study.

Between February and November 2010, 182 mothers were screened, of whom 104 were eligible for the study. Of the 102 deliveries, 94 infants were eligible for the study, including 79 breast feeders and 15 formula feeders (Fig. 1). At 20 weeks of age, 73 infants were randomized to receive the MVA.HIVA vaccine (*n* = 36) or no treatment (*n* = 37). Mothers of randomized infants had a median age of 27 years (IQR 22-31) and were enrolled at a median gestational age of 29 weeks (IQR 24, 32). The median infant birth weight was 3.1 kg (IQR 2.95, 3.4). Seventy-one infants completed visit 10 (48 weeks) within the scheduled visit window, with one infant attending late, giving an overall retention of 99% at 48 weeks. There were no significant differences between the 2 groups at baseline (Table 1).

### 3.2 MVA.HIVA was well tolerated

Most vaccinated infants had pain, redness and hardness on day 1 and 2 post-vaccination (Table 2). One week post-vaccination, 1 infant had grade 1 pain, 2 had redness measuring 0.3 and 0.5 cm and 14 had hardness with median (range) diameter of 0.5 (0.1–1) cm. All these events had resolved by 8 weeks post-vaccination. Three infants had lymphadenopathy measuring 0.5 cm in 2 infants and 0.6 cm in 1 infant at week 1; these resolved by week 8. Another infant had lymphadenopathy measuring 0.5 cm at week 8 (Table 2). As previously reported, 58% infants displayed hematologic toxicities pre-randomization [5]. However, there were no significant hematology or biochemistry differences between the vaccinees and controls post-vaccination (Table 3). There were 8 severe adverse events, 5 in the vaccine arm and 3 in the control arm. Among vaccinees, 1 infant had an upper respiratory tract infection, 2 had gastroenteritis, 1 had septicemia and 1 had a depressed skull fracture, while among controls, 2 infants had neutropenia and 1 had pneumonia (Table 4). None of these events were considered vaccine-related.

### 3.3 Vaccination did not induce significant, detectable anti-HIV-1 T-cell responses

A total of 262 *ex vivo* IFN-γ ELISPOT assays were conducted for 72 infants, with 18, 28, 14 and 12 infants tested at 5, 4, 3 and fewer time points, respectively. Results were also obtained for a total of 142 cultured assays from 51 infants with 39 and 12 infants tested at 3 and 2 time points, respectively. Overall, no positive HIV-1-specific T-cell responses were detected using either of the IFN-γ ELISPOT assays, although transiently higher frequencies were detected in the MVA.HIVA arm (*p* = 0.002) in fresh *ex vivo* assays, but not above the threshold frequencies considered as a positive result (Supplementary Table S1). Note, that infants have up to 15-fold higher PBMC counts per 1 ml of peripheral blood compared to adults.

### 3.4 Except for HBV, MVA.HIVA administration did not affect KEPI induction of protective antibodies

KEPI vaccinations elicited protective antibody levels to Hib, poliovirus, diphtheria, tetanus and pertussis in a majority of the infants with no statistically significant differences between the two arms (Table 5). For HBV, immune response to vaccine differed between the two groups; 71% of MVA.HIVA arm subjects versus 92% of control subjects achieved protective antibody levels to HBV (≥10 mIU ml^−1^) 1 week post-vaccination (*p* = 0.05), reflecting the greater drop in levels in the MVA.HIVA arm between weeks 19 and 21 (*p* = 0.025).

### 3.5 MVA.HIVA recipients remained HIV-1 test negative

Infants’ blood was regularly tested for HIV-1-specific DNA or antibodies. Post-randomization, all infants remained HIV negative at repeated serial testing.

## 4 Discussion

MVA is a boosting vector for recombinant subunit vaccines under development for prevention of a number of infectious diseases [14] for use in adults and infants [23,26,27]. The PedVacc 002 trial reported here demonstrated safety of MVA-vectored vaccines expressing an HIV-1-derived immunogen in 20-week-old HIV-1-negative African infants born to HIV-1-positive mothers.

Administration of one low MVA.HIVA vaccine dose without a heterologous prime or boost was not sufficiently immunogenic to induce HIV-1-specific, IFN-γ-producing T cells in the circulating blood of 20-week-old infants. There was also no indication of induction or boosting of infants’ HIV-1-specific T-cell responses through exposure to their mother's virus. This is neither unexpected nor discouraging for future use of this vaccine modality. First, because of the young age of vaccine recipients, we used a low intramuscular dose of MVA.HIVA, which was 4-fold lower than the adult dose of 2 × 10^8^ pfu [15] likely to be used in future studies. In addition, we and others have shown that vaccines vectored by MVA are poor primers of transgene-specific T-cell responses, but when given to well-primed individuals such as HIV-1-positive patients on ART or volunteers whose responses have already been expanded by DNA- and/or simian adenovirus-vectored vaccines, rMVA delivered up to a 10-fold boost to the existing frequencies of transgene-specific T-cells [15,28]. In our parallel PedVacc trials 001 and 002, this prudent rMVA vaccine dose was administered as the first stage of developing a recombinant BCG-MVA regimen with a possible extension to a dual HIV-TB vaccine platform [29–35]. Since the conception of these trials in 2007, both the immunogen design and its presentation to the immune system have evolved. Recently, a prime with non-replicating recombinant simian adenovirus followed by an rMVA boost was shown to induce high frequencies of transgene-specific T cells in UK adults [36–38]. The immunogen HIVA has been replaced by a pan-clade immunogen based on the most conserved regions of the HIV-1 proteome [36,39], which addresses virus diversity and escape more efficiently [28]. Furthermore, for a final vaccine regimen, an efficient T-cell vaccine will likely be combined with vaccines inducing broadly neutralizing antibodies when these become available [40].

MVA.HIVA did not interfere with responses to polio, diphtheria, pertussis, tetanus or Hib vaccines. However, a higher proportion of vaccinated infants failed to develop protective levels of antibodies to HBV. This difference was not observed in the PedVacc 001 study, where MVA.HIVA was administered to HIV-1-negative children of HIV-1-negative Gambian mothers and similar responses to the six childhood vaccines were found in vaccinees and controls [23]. A very good safety record of MVA.HIVA also concurs with candidate TB vaccine MVA85A, which was well tolerated in clinical trials in infants [26,27,41,42]. In the PACTG 326 and HPTN 027 studies, where non-replicating canarypoxviruses were administered to HIV-1-exposed uninfected (HEU) infants at birth, no differences in vaccine responses were found between infants and adults [20–22]. Nevertheless, immune system deficits that impair immune responses to childhood vaccines were described among HEU infants, not only resulting from abnormalities in the immune system [43–46], but also from antiretroviral prophylaxis administered to mothers for PMTCT [45]. Humoral vaccine responses among HEU infants were variable with similar responses being described 2 weeks after last vaccination [47] and lower HBV and tetanus titers 4 weeks after last vaccination [48] when compared to HIV-1-unexposed infants. In addition, HBV antibody level declined by up to 50% over time among HEU infants 6 months after the third vaccination dose emphasizing the need for boost vaccinations in this group [49]. Thus, reduced responses to HBV vaccine among HEU recipients of MVA.HIVA require further evaluation.

There was a high level of retention in this study despite the intensive study visits, demonstrating the feasibility of conducting vaccine studies among infants in the region. This finding is similar to other infant HIV-1 vaccine trials conducted in Africa [20–23] and provides reassurance for future vaccine evaluations in this age group.

In conclusion, MVA.HIVA was safe but not sufficiently immunogenic as a stand-alone vaccine in African infants. The safety profile demonstrated in PedVacc 001 [23] and 002 trials in infants, and immunogenicity of MVA-vectored vaccines observed in heterologous prime-boost regimens [29–35] support the use of MVA as a vaccine vector in infants. In addition to evaluating vaccine performance, both trials built capacity by using local ethics and regulatory review processes and establishing/expanding local infant HIV-1 vaccine trial expertise and facilities for evaluations of future vaccine candidates.

## Figures and Tables

**Table 1:** Characteristics of study participants.

	Control group (*n* = 37)	Vaccine group (*n* = 36)
Maternal characteristics at enrollment during pregnancy		
Maternal age (years)	27 (23, 31)	27 (22, 30.5)
Maternal CD4^+^ cell count (μl^−1^)	526 (428, 663)	549 (443, 658.5)
Gestational age (weeks)	28 (20,30)	30 (27,32)
Previously used ART	25 (67.6%)	23 (63.8%)
On AZT-based ART	16 (43.2%)	17 (47.2%)
Infant characteristics at 1 week before randomization (20 weeks postpartum)		
Male sex	16 (43.2%)	16 (44.4%)
Birth weight (g)	3,150 (3,000, 3,400)	3,125 (2,900, 3,405)
WAZ^a^	−0.66 (−1.22, 0.52)	−0.22 (−0.92, 0.71)
MUAC^b^ (cm)	14 (14, 15)	14.5 (14, 15)
Hemoglobin (gdl^−1^)	11.4 (10.9, 12.3)	11.85 (11.05, 12.3)
White Blood Cell count × 10^^3^^ (μl^−1^)	8.5 (7.6, 10.1)	9.85 (7.70,11.35)
Platelet count × 10^^3^^ (μl^−1^)	423 (308, 531)	428 (377.0, 514.5)
Neutrophil count × 10^^3^^ (μl^−1^)	1.35 (1.09, 1.59)	1.45 (1.12, 2.31)
Creatinine (μM)	37 (31, 43)	36.5 (31.5, 41)
Alanine transaminase (U l^−1^)	16 (12, 23)	19.5 (14.0, 25)

Values are given as median with interquartile range (IQR) in parentheses or *n* (%).

a Weight-for-age *Z*-score.

b Mid-upper arm circumference.

**Table 2:** Local reactogenicity during the first 8 weeks after vaccination.

*n* = 36	Day of vaccination	Day 1	Day 2	Week 1	Week 8
Pain	1	28	20	1	0
Grade 1	1	28	19	1	0
Grade 2	0	0	1	0	0
Grade 3	0	0	0	0	0
Redness	0	31	26	2^a^	0
Median (range) (cm)		0.2 (0.1–1)	0.3 (0.1–1.2)		
Hardness	0	17	20	14	0
Median (range) (cm)		0.4 (0.1–0.8)	0.4 (0.1–1)	0.5 (0.1–1)	
Ulceration	0	0	0	0	0
Lymph node^b^	0	1	0	3	1

a Two infants had redness at week 1 measuring 0.5 and 0.3 cm.

b One infant had lymphadenopathy noted at day 1 and week 1 at 0.6 cm. Two other infants had lymphadenopathy at week 1 measuring 0.5 cm. Another infant had lymphadenopathy measuring 0.5 cm at week 8. All events resolved spontaneously.

**Table 3:** Hematology and biochemistry data.

	Week 19		Week 21		Week 28	
	Vaccine	Control	Vaccine	Control	Vaccine	Control
Hemoglobin (g dl^−1^)	11.4 (10.4, 12.1)	11.2 (10.4, 12.3)	11.3 (10.8, 11.8)	11.1 (10.6, 12.4)	11.0 (10.3,11.7)	11.1 (10.7, 11.9)
White cell count × 10^3^ (μl^−1^)	9.5 (6.1, 11.0)	8.5 (6.3, 10.1)	10.4^a^ (7.5, 12.3)	8.7 (7.0, 11.3)	10.3^b^ (8.1, 12.6)	9.5 (7.8, 11.6)
Platelet count × 10^3^ (μl^−1^)	412 (290.5, 517.8)	319 (203.5, 524.5)	426 (289, 578.3)	471 (246, 576.5)	407 (290, 538)	464 (357, 556.5)
Neutrophil count × 10^3^ (μl^−1^)	1.3 (1.0, 2.2)	1.3 (1.0, 1.6)	1.7 (1.0, 2.3)	1.4 (1.0, 1.9)	1.5 (1.2, 2.4)	1.8^c^ (1.6, 2.9)
Creatinine (μM)	36.5 (31.3, 41.5)	37 (31, 43.5)	38.5 (33.2, 43)	39 (35, 42)	36 (33, 41)	37.5 (31.3, 42.5)
Alanine transaminase (U l^−1^)	19.5 (14, 25)	16 (11.5, 23.5)	18 (13.3, 26.5)	19 (14, 29)	18 (15, 23)	18.5 (14, 23)

Values are given as median (IQR).

a *p* = 0.08.

b *p* = 0.06.

c *p* = 0.08.

**Table 4:** Adverse events during the first 8 weeks after vaccination.

	Vaccine group (83 events)					Control group (72 events)				
		Grade					Grade			
	Total	1	2	3	4	Total	1	2	3	4
Systemic										
Upper respiratory tract infection	**22**	20	1	1	0	**16**	16	0	0	0
Gastroenteritis	**8**	6	0	2	0	**6**	6	0	0	0
Conjunctivitis	**3**	3	0	0	0	**2**	2	0	0	0
Cough	**4**	4	0	0	0	**0**	0	0	0	0
Fungal skin rash	**2**	2	0	0	0	**1**	1	0	0	0
Fever	**2**	2	0	0	0	**1**	1	0	0	0
Pneumonia	**1**	0	1	0	0	**1**	0	0	1	0
Rash	**3**	3	0	0	0	**0**	0	0	0	0
Otitis media	**0**	0	0	0	0	**1**	1	0	0	0
Septicemia	**1**	0	0	1	0	**0**	0	0	0	0
Eczema	**0**	0	0	0	0	**1**	1	0	0	0
Abscess	**0**	0	0	0	0	**1**	0	1	0	0
Colic	**0**	0	0	0	0	**1**	1	0	0	0
Depressed skull fracture	**1**	0	0	1	0	**0**	0	0	0	0
Constipation	**0**	0	0	0	0	**1**	1	0	0	0
Laboratory										
Elevated creatinine	**17**	15	2	0	0	**16**	13	3	0	0
Neutropenia	**12**	6	6	0	0	**12**	6	4	1	1
Anemia	**7**	6	1	0	0	**12**	7	5	0	0

**Table 5:** Antibody titres elicited by childhood vaccines one week post-vaccination.

EPI vaccine	Protective antibody level	Control week 21 median (min, max) *n*/total (%)	Vaccine week 21 median (min, max) *n*/total (%)	*p*—Difference between vaccine and control arm
Poliovirus	≥1:8	1024 (64, 1024)	1024 (128, 1024)	0.26
HBsAg (mIU ml^−1^)	≥10	55.0 (10, 1000)	27.0 (10, 1000)	0.32
HBV protective levels		24/26 (92%)	17/24 (71%)	0.05
Dtx (IU ml^−1^)	≥0.01	0.8 (0.08, 5.3)	0.8 (0.03, 3.5)	0.25
Ttx (IU ml^−1^)	≥0.01	3.2 (0.76, 9.0)	2.7 (0.16, 9.2)	0.35
Hib (μg ml^−1^)	≥0.15	6.4 (0.13, 190.3)	3.0 (0.08, 50.2)	0.29
Hib protective levels		25/26 (92%)	25/27 (93%)	0.51
Ptx (IU ml^−1^)	Unknown	39 (1, 437)	48 (1, 654)	0.96

## References

[1] Bae W.H., Wester C., Smeaton L.M., Shapiro R.L., Lockman S., Onyait K. (2008). Hematologic and hepatic toxicities associated with antenatal and postnatal exposure to maternal highly active antiretroviral therapy among infants. AIDS.

[2] Chappuy H., Treluyer J.M., Jullien V., Dimet J., Rey E., Fouche M. (2004). Maternal-fetal transfer and amniotic fluid accumulation of nucleoside analogue reverse transcriptase inhibitors in human immunodeficiency virus-infected pregnant women. Antimicrob Agents Chemother.

[3] Chappuy H., Treluyer J.M., Rey E., Dimet J., Fouche M., Firtion G. (2004). Maternal-fetal transfer and amniotic fluid accumulation of protease inhibitors in pregnant women who are infected with human immunodeficiency virus. Am J Obstet Gynecol.

[4] Feiterna-Sperling C., Weizsaecker K., Buhrer C., Casteleyn S., Loui A., Schmitz T. (2007). Hematologic effects of maternal antiretroviral therapy and transmission prophylaxis in HIV-1-exposed uninfected newborn infants. J Acquir Immune Defic Syndr.

[5] Njuguna I., Reilly M., Jaoko W., Gichuhi C., Ambler G., Maleche-Obimbo E. (2014). Infant neutrapenia associated with breastfeeding during maternal antiretroviral treatment for prevention of mother-to-child transmission of HIV. Retrovirology.

[6] Pacheco S.E., McIntosh K., Lu M., Mofenson L.M., Diaz C., Foca M. (2006). Effect of perinatal antiretroviral drug exposure on hematologic values in HIV-uninfected children: An analysis of the women and infants transmission study. J Infect Dis.

[7] Shapiro R.L., Holland D.T., Capparelli E., Lockman S., Thior I., Wester C. (2005). Antiretroviral concentrations in breast-feeding infants of women in Botswana receiving antiretroviral treatment. J Infect Dis.

[8] Six Week Extended-Dose Nevirapine Study, Bedri T.A., Gudetta B., Isehak A., Kumbi S., Lulseged S. (2008). Extended-dose nevirapine to 6 weeks of age for infants to prevent HIV transmission via breastfeeding in Ethiopia, India, and Uganda: an analysis of three randomised controlled trials. Lancet.

[9] Richardson B.A., Mbori-Ngacha D., Lavreys L., John-Stewart G.C., Nduati R., Panteleeff D.D. (2003). Comparison of human immunodeficiency virus type 1 viral loads in Kenyan women, men, and infants during primary and early infection. J Virol.

[10] Mo H., Monard S., Pollack H., Ip J., Rochford G., Wu L. (1998). Expression patterns of the HIV type 1 coreceptors CCR5 and CXCR4 on CD4+ T cells and monocytes from cord and adult blood. AIDS Res Hum Retroviruses.

[11] Luzuriaga K., Sullivan J.L. (2002). Pediatric HIV-1 infection: advances and remaining challenges. AIDS Rev.

[12] Farber D.L., Yudanin N.A., Restifo N.P. (2014). Human memory T cells: generation, compartmentalization and homeostasis. Nat Rev Immunol.

[13] Cunningham C.K., McFarland E. (2008). Vaccines for prevention of mother-to-child transmission of HIV. Curr Opin HIV AIDS.

[14] Gomez C.E., Perdiguero B., Garcia-Arriaza J., Esteban M. (2013). Clinical applications of attenuated MVA poxvirus strain. Expert Rev Vaccines.

[15] Hanke T., Goonetilleke N., McMichael A.J., Dorrell L. (2007). Clinical experience with plasmid DNA- and modified vaccinia vaccine Ankara (MVA)-vectored HIV-1 clade A vaccine inducing T cells. J Gen Virol.

[16] Hanke T., McMichael A.J. (2000). Design and construction of an experimental HIV-1 vaccine for a year-2000 clinical trial in Kenya. Nat Med.

[17] Borkowsky W., Wara D., Fenton T., McNamara J., Kang M., Mofenson L. (2000). Lymphoproliferative responses to recombinant HIV-1 envelope antigens in neonates and infants receiving gp120 vaccines. AIDS Clinical Trial Group 230 Collaborators. J Infect Dis.

[18] Cunningham C.K., Wara D.W., Kang M., Fenton T., Hawkins E., McNamara J. (2001). Safety of 2 recombinant human immunodeficiency virus type 1 (HIV-1) envelope vaccines in neonates born to HIV-1-infected women. Clin Infect Dis.

[19] McFarland E.J., Borkowsky W., Fenton T., Wara D., McNamara J., Samson P. (2001). Human immunodeficiency virus type 1 (HIV-1) gp120-specific antibodies in neonates receiving an HIV-1 recombinant gp120 vaccine. J Infect Dis.

[20] Johnson D.C., McFarland E.J., Muresan P., Fenton T., McNamara J., Read J.S. (2005). Safety and immunogenicity of an HIV-1 recombinant canarypox vaccine in newborns and infants of HIV-1-infected women. J Infect Dis.

[21] McFarland E.J., Johnson D.C., Muresan P., Fenton T., Tomaras G.D., McNamara J. (2006). HIV-1 vaccine induced immune responses in newborns of HIV-1 infected mothers. AIDS.

[22] Kintu K., Andrew P., Musoke P., Richardson P., Asiimwe-Kateera B., Nakyanzi T. (2013). Feasibility and safety of ALVAC-HIV vCP1521 vaccine in HIV-exposed infants in Uganda: results from the first HIV vaccine trial in infants in Africa. J Acquir Immune Defic Syndr.

[23] Afolabi M.O., Ndure J., Drammeh A., Darboe F., Mehedi S.-R., Rowland-Jones S.L. (2013). A phase I randomized clinical trial of candidate human immunodeficiency virus type 1 vaccine MVA.HIVA administered to Gambian infants. PLoS One.

[24] van Gageldonk P.G., van Schaijk F.G., van der Klis F.R., Berbers G.A. (2008). Development and validation of a multiplex immunoassay for the simultaneous determination of serum antibodies to Bordetella pertussis, diphtheria and tetanus. J Immunol Methods.

[25] Eggers M., Terletskaia-Ladwig E., Rabenau H.F., Doerr H.W., Diedrich S., Enders G. (2010). Immunity status of adults and children against poliomyelitis virus type 1 strains CHAT and Sabin (LSc-2ab) in Germany. BMC Infect Dis.

[26] Ota M.O., Odutola A.A., Owiafe P.K., Donkor S., Owolabi O.A., Brittain N.J. (2011). Immunogenicity of the tuberculosis vaccine MVA85A is reduced by coadministration with EPI vaccines in a randomized controlled trial in Gambian infants. Sci Transl Med.

[27] Scriba T.J., Tameris M., Mansoor N., Smit E., van der Merwe L., Mauff K. (2011). Dose-finding study of the novel tuberculosis vaccine, MVA85A, in healthy BCG-vaccinated infants. J Infect Dis.

[28] Hanke T. (2014). Conserved immunogens in prime-bost strategies for te next-generation HIV-1 vaccines. Expert Opin Biol Ther.

[29] Hopkins R., Bridgeman A., Bourne C., Mbewe-Mwula A., Sadoff J.C., Both G.W. (2011). Optimizing HIV-1-specific CD8^+^ T-cell induction by recombinant BCG in prime-boost regimens with heterologous viral vectors. Eur J Immunol.

[30] Hopkins R., Bridgeman A., Joseph J., Gilbert S.C., McShane H., Hanke T. (2011). Dual neonate vaccine platform against HIV-1 and M. tuberculosis. PLoS One.

[31] Im E.J., Saubi N., Virgili G., Sander C., Teoh D., Gatell J.M. (2007). Vaccine platform for prevention of tuberculosis and mother-to-child transmission of human immunodeficiency virus type 1 through breastfeeding. J Virol.

[32] Rosario M., Fulkerson J., Soneji S., Parker J., Im E.J., Borthwick N. (2010). Safety and immunogenicity of novel recombinant BCG and modified vaccinia virus Ankara vaccines in neonate rhesus macaques. J Virol.

[33] Rosario M., Hopkins R., Fulkerson J., Borthwick N., Quigley M.F., Joseph J. (2010). Novel recombinant *Mycobacterium bovis* BCG, ovine atadenovirus, and modified vaccinia virus Ankara vaccines combine to induce robust human immunodeficiency virus-specific CD4 and CD8 T-cell responses in rhesus macaques. J Virol.

[34] Saubi N., Gea-Mallorqui E., Ferrer P., Hurtado C., Sanchez-Ubeda S., Eto Y. (2014). Engineering new mycobacterial vaccine design for HIV–TB pediatric vaccine vectored by lysine auxotroph of BCG. Mol Ther Methods Clin Dev.

[35] Saubi N., Im E.J., Fernandez-Lloris R., Gil O., Cardona P.J., Gatell J.M. (2011). Newborn mice vaccination with BCG.HIVA + MVA.HIVA enhances HIV-1-specific immune responses: influence of age and immunization routes. Clin Dev Immunol.

[36] Borthwick N., Ahmed T., Ondondo B., Hayes P., Rose A., Ebrahimsa U. (2014). Vaccine-elicited human T cells recognizing conserved protein regions inhibit HIV-1. Mol Ther.

[37] Ewer K.J., O‘Hara G.A., Duncan C.J., Collins K.A., Sheehy S.H., Reyes-Sandoval A. (2013). Protective CD8(+) T-cell immunity to human malaria induced by chimpanzee adenovirus-MVA immunisation. Nat Commun.

[38] Sheehy S.H., Duncan C.J., Elias S.C., Choudhary P., Biswas S., Halstead F.D. (2013). ChAd63-MVA-vectored blood-stage malaria vaccines targeting MSP1 and AMA1: assessment of efficacy against mosquito bite challenge in humans. Mol Ther.

[39] Letourneau S., Im E.-J., Mashishi T., Brereton C., Bridgeman A., Yang H. (2007). Design and pre-clinical evaluation of a universal HIV-1 vaccine. PLoS One.

[40] McMichael A.J., Haynes B.F. (2012). Lessons learned from HIV-1 vaccine trials: new priorities and directions. Nat Immunol.

[41] McShane H., Pathan A.A., Sander C.R., Keating S.M., Gilbert S.C., Huygen K. (2004). Recombinant modified vaccinia virus Ankara expressing antigen 85A boosts BCG-primed and naturally acquired antimycobacterial immunity in humans. Nat Med.

[42] Tameris M.D., Hatherill M., Landry B.S., Scriba T.J., Snowden M.A., Lockhart S. (2013). Safety and efficacy of MVA85A, a new tuberculosis vaccine, in infants previously vaccinated with BCG: a randomised, placebo-controlled phase 2b trial. Lancet.

[43] Clerici M., Saresella M., Colombo F., Fossati S., Sala N., Bricalli D. (2000). T-lymphocyte maturation abnormalities in uninfected newborns and children with vertical exposure to HIV. Blood.

[44] Kidzeru E.B., Hesseling A.C., Passmore J.A., Myer L., Gamieldien H., Tchakoute C.T. (2014). In-utero exposure to maternal HIV infection alters T-cell immune responses to vaccination in HIV-uninfected infants. AIDS.

[45] Miyamoto M., Pessoa S.D., Ono E., Machado D.M., Salomao R., Succi R.C. (2010). Low CD4^+^ T-cell levels and B-cell apoptosis in vertically HIV-exposed noninfected children and adolescents. J Trop Pediatr.

[46] Nielsen S.D., Jeppesen D.L., Kolte L., Clark D.R., Sorensen T.U., Dreves A.M. (2001). Impaired progenitor cell function in HIV-negative infants of HIV-positive mothers results in decreased thymic output and low CD4 counts. Blood.

[47] Jones S.A., Groome M., Koen A., Van Niekerk N., Sewraj P., Kuwanda L. (2013). Immunogenicity of seven-valent pneumococcal conjugate vaccine administered at 6, 14 and 40 weeks of age in South African infants. PLoS One.

[48] Abramczuk B.M., Mazzola T.N., Moreno Y.M., Zorzeto T.Q., Quintilio W., Wolf P.S. (2011). Impaired humoral response to vaccines among HIV-exposed uninfected infants. Clin Vaccine Immunol.

[49] Thaithumyanon P., Punnahitananda S., Praisuwanna P., Thisyakorn U., Ruxrungtham K. (2002). Antibody response to hepatitis B immunization in infants born to HIV-infected mothers. J Med Assoc Thai.

