# Supplementary material for: PedVacc 002: A phase I/II randomized clinical trial of MVA.HIVA vaccine administered to infants born to human immunodeficiency virus type 1-positive mothers in Nairobi
Source: Vaccine. 2014 Oct 7;32(44):5801–8. doi: 10.1016/j.vaccine.2014.08.034 (PMC4414927; doi:10.1016/j.vaccine.2014.08.034)
Supplement: Supplementary file 1 [file mmc1.pdf]

Table S1. Fresh ex-*vivo* IFN-γ ELISPOT responses

		Week 19	Week 21	Week 28	Week 36	Week 48
Mock	**Vaccine**	0 (0, 1.7)	5 (0, 11.7)	1.7 (0, 3.3)	1.7 (0, 5)	0 (0, 5)
	**Control**	0 (0, 1.7)	0.8 (0, 2.5)	0 (0, 1.7)	0 (0, 1.7)	1.7 (0, 3.3)
PHA	**Vaccine**	635 (355, 1833)	770 (243, 2182)	858 (535, 1940)	1063 (710, 1953)	1323 (700, 2285)
	**Control**	1008 (438,1213)	505 (185.8, 1290)	443 (238, 1670)	1275 (750, 2110)	648 (282, 1768)
**PE**	**Vaccine**	0 (-1.7,.0)	0.8 (0, 3.3)	0 (-1.7, 3.3)	0 (-1.7, 3.3)	0 (0, 1.7)
	**Control**	0 (0, 2.5)[a]	0 (-1.7, 0)[b]	0 (-1.7, 1.7)	0 (0, 1.7)	0 (-1.7, 1.7)
**p24/p17**	**Vaccine**	0 (-1.7, 1.7)	1.7 (-1.7, 6.7)	-0.8 (-1.7,1.7)	0 (-1.7, 1.7)	0 (-1.7, 1.7)
	**Control**	0 (0, 0)	0 (-1.7, 0)[c]	0 (-1.7, 0)	0 (-1.7, 0)	0 (-1.7, 0)
**PE+p24/p17**	**Vaccine**	0 (-3.3, 1.7)	4.2 (0, 8.3)	0 (-3.3, 5)	0 (-3.3, 5)	0 (-1.7, 3.3)
	**Control**	0 (0, 3.3)	0 (-1.7, 0)[d]	0 (-1.7, 1.7)	0 (0, 2.5)	0 (-3.3, 1.7)

a - p = 0.053; b - p = 0.069; c - p = 0.035; d - p = 0.002

Median values expressed as SFU/10^6 PBMC are shown with IQR in brackets. Responses to the polyepitope (PE) and Gag p24/p17 regions of the HIVA immunogen, and the PHA positive control are reported as net values, i.e. after subtraction of background. The raw no-peptide "Mock" background values are also shown. Note, that infants and adults have typically 15 x 10^6 and 1 x 10^6 PBMC per 1 ml of peripheral blood, respectively.
