# Supplementary material for: PedVacc 002: A phase I/II randomized clinical trial of MVA.HIVA vaccine administered to infants born to human immunodeficiency virus type 1-positive mothers in Nairobi
Source: Vaccine. 2014 Oct 7;32(44):5801–8. doi: 10.1016/j.vaccine.2014.08.034 (PMC4414927; doi:10.1016/j.vaccine.2014.08.034)
Supplement: Supplementary file 3 [file mmc3.pdf]

AN OPEN RANDOMIZED PHASE I/II STUDY EVALUATING SAFETY AND IMMUNOGENICITY OF A CANDIDATE HIV-1 VACCINE, MVA.HIVA, ADMINISTERED TO HEALTHY INFANTS BORN TO HIV-1-INFECTED MOTHERS

PV002 CLINICAL TRIAL PROTOCOL

Sponsor: Medical Research Council (MRC) UK
OXTREC Reference No: 52 08
Kenyatta Ethics No: P266/10/2008
Seattle Ethics No: 32762

University of Nairobi Principal Investigator: Prof. Walter Jaoko

Version Number: 3
July 6, 2009

SIGNATURE PAGE

The signature below constitutes the approval of this protocol and the attachments, and provides the necessary assurances that this trial will be conducted according to all stipulations of the protocol, including all statements regarding confidentiality, and according to local legal and regulatory requirements and applicable UK regulations and ICH guidelines.

Chief Investigator:

Signed: ______________________ Date: 23/6/2009

Tomas Hanke, BSc, MSc, PhD

Principal Investigator:

Signed: ______________________ Date: 21/06/2009

Walter Jaoko, MB; ChB, MTropMed; PhD

Sponsor:

Signed: ______________________ Date: 24-6-09

Medical Research Council, UK

TABLE OF CONTENTS

		Page
**1**	**KEY ROLES**	**9**
**2**	**BACKGROUND INFORMATION AND SCIENTIFIC RATIONALE**	**10**
**2.1**	**The need for an HIV-1 vaccine against mother-to-child transmission**	**10**
2.1.1	MVA.HIVA design.	11
2.1.2	Pre-clinical studies with MVA-vectored recombinant vaccines.	11
2.1.3	Pre-clinical safety and immunogenicity of MVA.HIVA.	12
2.1.4	Safety of recombinant MVA vaccines in humans.	12
2.1.5	Safety of MVA.HIVA in adult humans.	12
2.1.6	Clinical Trials with the MVA.HIVA Vaccine.	13
2.1.7	Containment and transmission of live MVA-vectored vaccines.	14
**2.2**	**Potential Risks and Benefits**	**14**
2.2.1	Potential Risks	14
2.2.2	Known Potential Benefits	14
**3**	**OBJECTIVES**	**15**
**4**	**STUDY DESIGN**	**16**
**4.1**	**Sample size:**	**16**
**4.2**	**Randomization:**	**17**
**5**	**STUDY POPULATION**	**18**
**5.1**	**Inclusion Criteria**	**18**
**5.2**	**Exclusion Criteria**	**19**
**5.3**	**Sensitisation and Recruitment Procedures**	**20**
**6**	**STUDY ENROLMENT AND SCHEDULE OF FOLLOW-UP VISITS**	**20**
**6.1**	**Definitions**	**23**
**6.2**	**Study Visits:**	**23**
**7**	**STUDY INTERVENTION / INVESTIGATIONAL PRODUCT**	**27**
**8**	**ASSESSMENT OF SCIENTIFIC OBJECTIVES**	**28**
**9**	**ASSESSMENT OF SAFETY**	**28**
**9.1**	**Definitions**	**29**
**9.2**	**Adverse Events Assessment**	**30**
**9.3**	**Adverse Event Monitoring**	**31**
**10**	**CLINICAL MONITORING STRUCTURE**	**32**
**11**	**STATISTICAL CONSIDERATIONS**	**33**

11.1 Determination of the Sample Size 33

11.2 Expected analyses and outcomes 34
11.2.1 Safety of MVA.HIVA 34
11.2.2 Immunogenicity of MVA.HIVA 34
11.2.3 Immunogenicity of KEPI vaccines 34

11.3 Data Management 34

12 SOURCE DOCUMENTS AND ACCESS TO SOURCE DATA/DOCUMENTS 34

13 QUALITY CONTROL AND QUALITY ASSURANCE 35

14 ETHICS/PROTECTION OF HUMAN SUBJECTS 35

14.1 Informed Consent Process 35

14.2 Subject Confidentiality 35

14.3 Biohazard Containment 36

15 DATA HANDLING AND RECORD KEEPING 36

16 PLANS FOR DISTRIBUTION OF RESEARCH FINDINGS TO STUDY COMMUNITY 37

16.1 Community Advisory Group 37

17 REFERENCES 37

18 APPENDIX 1 - CLINICAL TRIAL INFORMATION SHEET 41

19 APPENDIX 2 - INFORMED CONSENT FORM 44

LIST OF ABBREVIATIONS

**3TC**	Lamivudine
**AIDS**	Acquired immunodeficiency syndrome
**AE**	Adverse event
**ALT**	Alanine aminotransferase
**ARV**	Antiretroviral
**AZT**	Azidothymidine (also known as zidovudine)
**BD**	Bi-daily
**BF**	Breastfeeding
**BCG**	Bacillus Calmette-Guérin vaccine (against tuberculosis)
**CCC**	Comprehensive Care Clinic
**CRF**	Case Report Form
**CTL**	Cytotoxic T lymphocyte
**DAIDS**	Division of AIDS
**DMEC**	Data Monitoring & Ethics Committee
**DTP**	Diphtheria, tetanus and pertussis vaccine
**ELISA**	Enzyme-linked immunosorbant assay
**ELISPOT**	Enzyme-linked immunospot
**FBC**	Full blood count
**FF**	Formula-feeding
**GCP**	Good Clinical Practice
**GBS**	Guillain-Barré syndrome
**GMO**	Genetically modified organism
**GMP**	Good Manufacturing Practice
**HAART**	Highly active antiretroviral treatment
**HBV**	Hepatitis B virus vaccine
**HiB**	Haemophilus influenzae type b vaccine
**HIV**	Human immunodeficiency virus
**HLA**	Human leukocyte antigen
**ICH**	International Conference on Harmonization
**IFN-γ**	Gamma interferon
**KEPI**	Kenyan Extended Programme on Immunization
**KNH**	Kenyatta National Hospital
**LPV**	Lopinavir
**LSM**	Local safety monitor
**MTCT**	Mother-to-child transmission
**MUAC**	Mid-left upper arm circumference
**MVA.HIVA**	Recombinant non-replicating modified vaccinia virus Ankara expressing HIV-1-derived immunogen HIVA
**NAb**	Neutralizing antibodies
**OPV**	Oral polio vaccine
**OXTREC**	Oxford Tropical Research Ethics Committee
**PBMC**	Peripheral blood mononuclear cells

**PI**	Principal Investigator
**PMTCT**	Prevention of mother-to-child transmission
**SAE**	Serious adverse event
**SIV**	Simian immunodeficiency virus
**SOP**	Standard operating procedure
**SUSAR**	Suspected unexpected serious adverse reactions
**TSC**	Trial Steering Committee

PROTOCOL SUMMARY

Title:
An open randomized phase I/II study evaluating safety and immunogenicity of a candidate HIV-1 vaccine, MVA.HIVA, administered to healthy infants born to HIV-1-infected mothers.

Phase:
Phase I/II

Population:
N = 72 HIV-1 negative infants:
- 36 breastfeeding infants, 18 randomized to MVA.HIVA and 18 randomized age-matched unvaccinated controls
- 36 formula feeding infants, 18 randomized to MVA.HIVA and 18 randomized age-matched unvaccinated controls

Number of Sites:
One: Kenyatta National Hospital, Nairobi, Kenya

Study Duration:
15 months per subject

Description of Investigational Product/Intervention:
1 dose of 5×10^7 pfu of MVA.HIVA administered intramuscularly

Blinding: There will be no blinding in the field, but the samples will be coded and the randomisation group will not be known to the laboratory staff performing and analysing the immunological assays.

Objectives:
Primary: Safety and immunogenicity of MVA.HIVA vaccine in 20-week-old healthy Kenyan infants born to HIV-1-infected mothers.

Secondary:
- HIV-1 immunogenicity comparison between MVA.HIVA and age-matched unvaccinated control arms in each cohort (breastfeeding or formula feeding)
- HIV-1 immunogenicity comparison between breastfeeding and formula feeding infants receiving MVA.HIVA
- HIV-1 immunogenicity comparison between breastfeeding and formula feeding infants in the age-matched unvaccinated control group
- Comparison of responses to certain Kenyan Extended Programme on Immunization (KEPI) vaccines (OPV, DTP, HBV, and HiB) between MVA.HIVA versus age-matched unvaccinated controls in each cohort, between breast versus formula feeding infants in the age-matched unvaccinated control group, and between breast versus formula infants receiving MVA.HIVA
- Comparison of immune activation and phenotypic profile of lymphocytes between breast and formula feeding infants in each cohort (MVA.HIVA and age-matched unvaccinated control)
- Build capacity for Infant HIV-1 Vaccine Clinical Trials Centre in Nairobi, Kenya.

Description of Study Design:
This is an open randomised phase I/II study of a candidate HIV vaccine, MVA.HIVA, in healthy, HIV-1 negative infants born to HIV-1 infected mothers. The immunology lab will be blinded for the investigational product/control allocation.

1 KEY ROLES

For questions regarding this protocol, contact:

Professor Walter Jaoko, MB, ChB, MTropMed, PhD, Site Principal Investigator
Department of Medical Microbiology
University of Nairobi
Second Floor, PO Box 19676
Off Ngong Road, Nairobi
Kenya
Phone: +254-02-2717694 / 275404
Email: Wjaoko@kaviuon.org

Tomas Hanke, BSc, MSc, PhD, Chief Investigator
Weatherall Institute of Molecular Medicine
University of Oxford
The John Radcliffe
Oxford OX3 9DS
Phone: +44 (0)1865 222355
Email: tomas.hanke@imm.ox.ac.uk

Institutions:

Sponsor:	Medical Research Council, 20 Park Crescent, London W1B 1AL
Local Trial Monitor:	This study will be independently monitored by Joyce Katuu, ProclinKenya Limited
Study sites:	Kenyatta National Hospital, Nairobi, Kenya
Clinical laboratory:	University of Nairobi, Nairobi, Kenya
Local Safety Monitor:	Day to day safety oversight will be provided by Jacquelyn Nyange, Kenya AIDS Vaccine Initiative (KAVI)

Co-Chairs for Protocol Development:

Grace John-Stewart	University of Washington	Associate Professor
Walter Jaoko	University of Nairobi	Associate Professor
Dorothy Mbori-Ngacha	University of Nairobi	Associate Professor

Investigators:

Tomas Hanke	University of Oxford	Reader
Walter Jaoko	University of Nairobi	Associate Professor
Grace John-Stewart	University of Washington	Associate Professor
Andrew McMichael	University of Oxford	Professor
Barbara Lohman Payne	University of Washington	Assistant Professor
Omu Anzala	University of Nairobi	Associate Professor
Dalton Wamalwa	University of Nairobi	Senior Lecturer
Ruth Nduati	University of Nairobi	Associate Professor
Marie Reilly	Karolinska Institute	Professor
Dorothy Mbori-Ngacha	University of Nairobi	Associate Professor
Elizabeth Obimbo	University of Nairobi	Senior Lecturer
Carey Farquhar	University of Washington	Assistant Professor
Barbra Richardson	University of Washington	Associate Professor

2 BACKGROUND INFORMATION AND SCIENTIFIC RATIONALE

2.1 The need for an HIV-1 vaccine against mother-to-child transmission

Since the first report of AIDS in 1981, it is estimated that over 60 million people have become infected with HIV-1, of who some 25 million have died. Over 60% of the global HIV-1-infected population lives in Africa and about half of the infected adults are women of child-bearing age. From 10 to 40% of pregnant women attending urban maternal child health clinics in Kenya are HIV-1 seropositive. Despite the fact that approximately half of mother-to-child transmission (MTCT) is due to prolonged breast-feeding [1], for many HIV-1-infected mothers formula feeding is not an option for social, practical and health reasons. Formula-fed babies of infected mothers have a higher morbidity and mortality due to increased exposure and susceptibility to other infections [2]. Although antiretroviral therapy can significantly reduce the risk of MTCT at parturition, it is less clear whether it is practical to use these drugs for extended periods to prevent HIV-1 transmission by breast milk. The drugs are expensive and treatment has to be maintained throughout the whole period of breastfeeding; furthermore the drugs' effectiveness might be compromised by emergence of resistant mutants. Thus, one of the best hopes for protecting newborns and infants in developing countries against MTCT of HIV-1 is the through the development of a safe, effective, accessible prophylactic vaccine, which could both reduce the adult burden of infection and/or protect neonates against vertical HIV-1 transmission.

There is no doubt that a vaccine inducing both neutralizing antibodies (NAb) and T cells will have the best chance to benefit vaccinees. However, development of vaccines inducing broadly neutralizing antibodies against HIV-1 has proven impossible so far and is clearly extremely difficult to achieve. The alternative approach exemplified by many HIV-1 vaccines currently in trials focuses on the induction of T cell immunity. T cells function by killing HIV-1-infected cells and producing soluble factors that can directly and indirectly control HIV-1 spread. While for prophylactic vaccination T cells cannot prevent the first wave of transmitted virus from infecting host cells, they could limit the extent of early viral infection and are expected to increase the dose of incoming HIV-1 necessary to establish infection. As a result, potent vaccine-induced HIV-1-specific T cell responses could decrease tissue damage during the acute phase of infection and improve the control of the virus load leading to a lower set point, thus delaying the development of AIDS and reducing viral transmission. The current T cell vaccination protocols can achieve a partial protection in non-human primate challenge models, in which animals are infected with higher doses of and more pathogenic viruses than those encountered by humans. Therefore there is a good case for believing that induction of T cell responses by vaccination will translate into a real benefit for human recipients. In animal models, qualitative differences in memory T cells may dramatically influence vaccine-mediated protective immunity even when the frequencies of HIV-1-specific T cells are similar [3]. Thus, to test the T cell hypothesis adequately, means of inducing high frequencies of multifunctional, durable T cells with strong proliferative potential and recognizing protective rather than immunodominant epitopes will need to be developed. Recent discontinuation of vaccination and enrolment in phase IIb trials of Merck's human adenovirus serotype 5 (MRK rAd5)-vectored vaccine should be noted, but it is not a reason for general dismissal of the T cell strategy. Both the Merck vaccine delivery vector and HIV-1-derived immunogens can be improved on and thus the results of these trials are regarded as a 'product' rather than 'concept failure'. Clearly, more vaccine concepts need to be tested in phase IIb test-of-principle trials. Furthermore, once a NAb-inducing vaccine becomes available, this can be combined with the best performing T cell vaccine for maximum benefit.

It is not known how early in life T cells can be educated to launch a protective response against intracellular microorganisms and this most likely differs from pathogen to pathogen. Qualitative and quantitative differences between responses in human newborns and adults are observed for a number of infections [4-7]. In HIV-1-infected infants, lower CD8 T cell responses compared to adults may play an important role in the faster disease

progression [8-10]; children account for 4% of HIV-1 infections, yet they represent 20% of AIDS deaths [5]. At the same time, mature responses to certain infections and vaccines have been demonstrated during the postnatal and even foetal period [11]. This is particularly true for BCG vaccine-induced responses, which promote adult-like Th1 response in newborns [12-15]. Therefore, there is some evidence suggesting that protective T cell-mediated responses could be elicited by vaccines in early life, and BCG as a vaccine vector might be very well suited to prime them [16].

Taken together, it is our hypothesis that priming HIV-1-specific responses as early in life as possible, that is by using BCG.HIVA, can decrease the transmission of HIV-1 from infected mothers to infants. These BCG.HIVA-primed responses can be boosted by MVA.HIVA and/or through natural exposure to HIV-1 in breast milk. Furthermore, the MVA.HIVA boost can be combined with MVA.85A to also increase the protection against TB [17]. Thus, this approach can serve as a vaccine platform providing infants a dual protection against both TB and HIV-1. The Kenyatta Clinical Trial Protocol herein is the first phase of the overall effort towards evaluation of the BCG.HIVA-MVA.HIVA prime-boost regimen in infants born to HIV-1-infected mothers. The Kenyatta Clinical Trial will test safety and immunogenicity of MVA.HIVA alone in infants born to HIV-1-infected mothers and any possible gross interference of the MVA.HIVA with the vaccines of the KEPI. The trial will not be powered to detect small interference effects.

2.1.1 MVA.HIVA design.

The live attenuated MVA.HIVA is a recombinant MVA that contains the coding sequence of immunogen HIVA. The HIVA immunogen consists of consensus HIV-1 clade A gag p24/p17 sequences and a string of $CD8^+$ T cell epitopes, including those recognized by mouse and rhesus macaque T cells, and a monoclonal antibody epitope tag [18]. The non-recombinant MVA was provided by Professor A. Mayr. The recombinant MVA was constructed by double recombination, using the β-galactosidase gene as marker. The HIVA is expressed from the late viral promoter P7.5 of vaccinia virus.

The relevance of the clade A HIV-1-derived immunogen HIVA for the viruses currently circulating in Africa was demonstrated by detection of positive responses in an IFN-γ ELISPOT assay using either MVA.HIVA-infected or HIVA peptide-pulsed peripheral blood mononuclear cells (PBMC) in HIV-1-infected and exposed uninfected infants in Nairobi [19]. The utility of the HIVA immunogen was also shown by boosting HIV-1-specific responses in individuals in Oxford infected with diverse HIV-1 clades [20, 21].

2.1.2 Pre-clinical studies with MVA-vectored recombinant vaccines.

The immunogenicity of MVA-based recombinant vaccines in animals is well documented. Recombinant MVA vector vaccines alone or in combination with other vectors elicit insert-specific cytotoxic T lymphocytes (CTL) responses in mice [22-24] and rhesus monkeys [25-27], decrease plasma viraemia and increase survival [27, 28] after challenge with pathogenic simian immunodeficiency virus (SIV). Several studies in macaques have demonstrated that, although CTL responses were detected following immunization with MVA-SIV recombinants, no animals were completely protected from infection upon challenge with pathogenic SIV. However, vaccinated animals had lower virus loads and prolonged survivals compared with control animals that received only non-recombinant MVA [27-29].

The safety of recombinant MVA was studied in immune-suppressed macaques. Eight macaques were vaccinated with MVA by three different routes (intradermally, intramuscularly and intranasally) after immune-suppression by total body irradiation, anti-thymocyte globulin treatment or measles virus infection. No clinical, haematological or pathological abnormalities related to MVA inoculation were observed during a 13-day follow-up period [30].

2.1.3 Pre-clinical safety and immunogenicity of MVA.HIVA.

The MVA.HIVA vaccine has been developed as a component of a DNA prime-recombinant MVA boost regimen. Pre-clinical safety studies with the pTHr.HIVA DNA and MVA.HIVA vaccines were carried out in compliance with Good Laboratory Practice at Huntingdon Life Science, UK. A combined protocol of persistence, distribution and toxicity of the pTHr.HIVA and MVA.HIVA vaccines in the BALB/c mouse was performed, which demonstrated that the vaccines were non-toxic and detectable beyond 5 weeks after administration only in the sites of injection [31]. These results formed a basis for the approval of phase I safety and immunogenicity clinical trials in healthy HIV-1-uninfected volunteers. To support studies in HIV-1-infected subjects, toxicity and bio-distribution of MVA.HIVA in mice with severe combined immunodeficiency (SCID) and SIV-infected rhesus macaques were carried out, which demonstrated that the MVA.HIVA vaccine was non-toxic in mice and non-persistent in places other than the injection site in mice and monkeys [32].

T cell immunogenicity of both the pTHr.HIVA and MVA.HIVA vaccines alone and in prime-boost combinations have been demonstrated extensively in mice [18, 31, 33-38] and rhesus macaques [26, 32, 37, 39].

2.1.4 Safety of recombinant MVA vaccines in humans.

Poxviruses have played an important role in the field of vaccinology ever since Edward Jenner in 1798 protected humans against smallpox (variola) by inoculation of the related cowpox virus. MVA, a highly attenuated strain of vaccinia virus and a member of the *Orthopoxvirus* genus in the family of *Poxviridae*, made its debut towards the end of the smallpox eradication campaign as a safer alternative vaccine and has a good safety record [40]. It has been administered to more than 120,000 vaccinees as part of the smallpox eradication programme, with no reported adverse effects, despite the deliberate vaccination of high risk groups [41]. There are now more safety data from a number of recombinant MVA based vaccines expressing antigens for hepatitis B, malaria, HIV, and melanoma from phase I/II trials both in the UK and Africa [42-44].

Recombinant subunit vaccines vectored by non-replicating poxvirus MVA have an impressive record of safety from HIV-1/2-uninfected adults [45-47], children [42, 48-50] and infants (McShane, personal communication). This is further supported by an excellent safety record of closely related non-replicating recombinant poxviruses NYVAC, ALVAC and fowlpox mostly in adults [51-55], but also in children [42, 48-49] and infants [56].

Preliminary data from MVA85A vaccine [17] tested in infants in Sukuta for its immunogenicity and interference with other EPI vaccine suggest that it is safe and immunogenic (Martin Ota, personal communication). However, a preliminary analysis shows that co-administration of MVA85A with the third dose of DTP, HiB, HBV caused a borderline decrease in the number of children with protective levels of HiB antibodies (>0.5) compared to those who received EPI alone ($p = 0.06$). This effect was no longer apparent when a cutoff for protective antibodies of >1.0 was used, in line with the UK National Vaccine Evaluation Consortium value for protective levels ($p = 0.39$). Antibody responses to diphtheria, tetanus and hepatitis B were not affected by MVA85A. As a result of these data it has been decided not to administer MVA.HIVA simultaneously with KEPI vaccines in this study.

2.1.5 Safety of MVA.HIVA in adult humans.

The complete list of clinical studies using the MVA.HIVA vaccine is given in Table 1. All these trials were performed prior to the introduction of the EudraCT numbering. In summary, the MVA.HIVA vaccine was used in 13 phase I and II preventive clinical trials and some therapeutic studies [57], in which 375 subjects received 741 doses of MVA.HIVA at four dosage levels ranging from 5×10^6 to 2.5×10^8 plaque-forming units using three routes of administration (intradermally, intramuscularly, and sub-cutaneously). In some studies, MVA.HIVA was given alone or as boost to pTHr.HIVA DNA. No SAEs definitely or probably

related to rMVA.HIVA have been reported. In healthy subjects, the short- and medium-term safety profiles in these studies indicate that the MVA.HIVA vaccine is generally safe and well tolerated. Following intradermal vaccination, local redness and discomfort were frequently reported, with a few vaccinees developing a crust and scab. Reactogenicity peaked at 3 days post-vaccination, resolved spontaneously by 2 weeks and was milder for a second dose of the vaccine. One volunteer in the IAVI 003 trial experienced an SAE 2 days after the first vaccination, which involved fever of 38.5 ^{o}C, malaise, myalgia, nausea and headache. This volunteer was hospitalised for 2 days for observation and rehydration. The fever and vomiting resolved within 48 hours and the malaise 2 days later. The SAE fully resolved 10 days after onset. The event was judged as possibly related to the vaccine and this volunteer did not receive the second dose of MVA.HIVA. The same volunteer had similar complaints again 2 months later despite discontinued vaccination. During this second event, several individuals had similar complaints at the hospital where the volunteer worked as a nurse and investigations suggested an epidemic of a viral infection caused by the small round-structure virus (SRSV). The relationship between the second adverse event and the vaccine remains unclear [58]. Vaccination of HIV-1-infected patients on highly active antiretroviral treatment (HAART) with MVA.HIVA was well tolerated and no serious adverse events were observed. $CD4^+$ T cell counts remained stable and viral loads were undetectable throughout the follow up, which was for one year after the first immunization [20, 21]. It should be noted that in most of these studies the MVA.HIVA vaccine was administered intradermally, but in the proposed Clinical Protocol PV002 it will be delivered intramuscularly. This is expected to decrease the local skin reactivity.

Table 1. Completed trials involving MVA.HIVA vaccination.

Trial No.	Location	No. Subjects Receiving MVA.HIVA	HIV Status	Total No. of Doses in Trial
IAVI 003	Oxford	8	-	15
IAVI 005	Oxford	9	-	18
IAVI 004	Nairobi	12	-	21
IAVI 002/8	Nairobi	10	-	20
IAVI 006	Oxford/St. Mary's, London	108	-	216
IAVI 009	Uganda	40	-	80
IAVI 010	Nairobi/St. Thomas, London	90	-	180
IAVI 011	Lausanne/PHRU-Soweto/MRC Durban/Simbec-Wales	66	-	132
IAVI 016	Oxford	16	-	24
Ther 002	Oxford	8	+	16
Ther 003/4	Oxford	8	+	19
**Total**		**375**		**741**

Data from MVA.HIVA vaccine [17] tested in infants in The Gambia for its immunogenicity and interference with other KEPI vaccine will be available in early 2010.

2.1.6 Clinical Trials with the MVA.HIVA Vaccine.

MVA.HIVA vaccine delivered alone and in a prime-boost regimen was tested in several hundred healthy and HIV-1-infected volunteers in Europe and Africa, and the clinical immunogenicity results are summarized in a recent publication [57]. Overall, these trials demonstrated that MVA.HIVA vaccine is less efficient in priming HIV-1-specific responses, but it can deliver a consistent boost to both $CD4^+$ and $CD8^+$ T cells, which is particularly strong if the T cell responses are well primed, e.g., HIV-1 infected patients [20, 21, 59]. Thus, in the most recent trial in healthy volunteers, MVA.HIVA alone primed responses were

not detectable using the *ex vivo* IFN-γ ELISPOT assay, but in cultured IFN-γ ELISPOT assays, HIV-1-specific memory T cells were found in 5 out of 8 volunteers. When primed with a pTHr.HIVA DNA vaccine, MVA.HIVA induced detectable memory HIV-1-specific predominantly $CD4^+$ T cell responses in 8 out of 8 volunteers [60]. In HIV-1 infected patients on HAART, a significant amplification and broadening of $CD8^+$ and $CD4^+$ IFN-γ responses to vaccine-derived epitopes was observed in all (n=16) vaccinees, but not in unvaccinated controls, in the absence of rebound viraemia. After MVA.HIVA administration, vaccine-expanded $CD8^+$ T cells identified by tetramer reactivity were transiently activated and had upregulated perforin levels. Expansions persisted for at least one year and consisted predominantly of either $CD45RA^-$ $CCR7^+$ or $CD45RA^-$ $CCR7^-$ $CD8^+$ T cells. Increased frequencies of $CD4^+$ T cells expressing intracellular IL-2 and IFN-γ were noted in several vaccinees [21].

2.1.7 Containment and transmission of live MVA-vectored vaccines.

The live MVA vector does not replicate in human cells and therefore is not transmissible between individuals. To prevent leakage of recombinant MVA following intramuscular injection, the injection site will be covered with a dry adhesive dressing 10 minutes after the vaccination for at least 50 minutes.

The UK Health and Safety Executive (HSE) has previously classified MVA.HIVA as a Class I Genetically Modified Organism (GMO). A standard operating procedure (SOP) regarding the contained use of GMOs has been developed following recommendations of the Gene Therapy Advisory Committee (GTAC), UK.

2.2 Potential Risks and Benefits

2.2.1 Potential Risks

It is important to note that MVA has been administered to over 120,000 subjects as part of the smallpox campaign with no reported adverse events, and therefore has a good safety record. The general risks to participants in this phase I/II study are associated with phlebotomy and with vaccination. The total volume of blood drawn over the study period (up to 25 ml) should not compromise these otherwise healthy infants. Potential risks include the following:

Local Reactions: Mild tenderness and bruising may result from venepuncture. An inflammatory reaction as manifested by redness, swelling, scaling and/or tenderness may occur at the site of vaccine injection. In previous studies with MVA.HIVA, these local reactions resolved spontaneously within a couple of days.

Systemic Signs and Symptoms: Systemic events following administration of MVA as a vector that could potentially occur include a flu-like illness with low-grade fever, chills and malaise. As with any other vaccine, temporary ascending paralysis, the Guillain-Barré syndrome (GBS) or immune mediated reactions that can lead to organ damage may occur. For influenza vaccines an excess of approximately 1 GBS case per million persons immunized has been observed. No cases were observed in people under 45 years of age. However, GBS has never been known to be caused by MVA.HIVA or vaccines containing any of their components.

Allergic Reactions and Anaphylaxis: As with any vaccine, allergic reactions are possible. All subjects will be closely monitored and followed up.

2.2.2 Known Potential Benefits

This is a preliminary study with the long-term goal of proving that a vaccine can protect against mother-to-child transmission of HIV-1 infection in humans. There are no proven data to suggest that infants will benefit directly from participation in this study by being protected

against HIV-1 via the MVA.HIVA vaccine. However, it is hoped that the information gained from this study will contribute to the development of a safe and effective HIV-1 vaccine for prevention of MTCT of HIV-1 through breast milk, and thus long term foreseeable benefits for these children when they reach child bearing age and for their children can be envisaged.

All participants will receive their Kenyan Expanded Programme on Immunization (KEPI) vaccines and free medical health care at Kenyatta National Hospital (KNH) during their participation in the study, including the cost of delivery and antenatal care visits at KNH. All participants will have access to free antiretrovirals to prevent mother-to-child transmission during pregnancy and delivery, and those women who decide to breastfeed their infants will also recieve free antiretrovirals during the period of breastfeeding. Participants will be followed up at home and in the clinic by one of the study field workers and/or nurses. Participants will therefore benefit by having information about their general health status, and the rigorous follow up visits could also enhance early detection and management of medical conditions that might arise in the course of the study. Any infant with antibody titres to KEPI vaccines below the level of protection will receive a booster dose.

All mothers will have access to regular counselling regarding their feeding choice and practices, and those women who decide to formula feed their infants will also receive formula free of charge.

3 OBJECTIVES

Primary:

- Safety and immunogenicity of MVA.HIVA vaccine in healthy (HIV-negative) Kenyan infants born to HIV-1-infected mothers.

Secondary:

- HIV-1 immunogenicity comparison between MVA.HIVA and age-matched unvaccinated control arms in each cohort (breastfeeding or formula feeding)
- HIV-1 immunogenicity comparison between breastfeeding and formula feeding infants receiving MVA.HIVA
- HIV-1 immunogenicity comparison between breastfeeding and formula feeding infants in the age-matched unvaccinated control group
- Comparison of responses to KEPI vaccines (OPV, DTP, HBV, and HiB) between MVA.HIVA versus age-matched unvaccinated controls in each cohort, between breast versus formula feeding infants in the age-matched unvaccinated control group, and between breast versus formula infants receiving MVA.HIVA
- Comparison of immune activation and phenotypic profile of lymphocytes between breast and formula feeding infants in each cohort (MVA.HIVA and age-matched unvaccinated controls)
- Build capacity for Infant HIV-1 Vaccine Clinical Trials Centre in Nairobi, Kenya.

Endpoints:

<u>For safety and reactogenicity:</u> Actively and passively collected data on adverse events (AEs).

<u>For immunogenicity to KEPI vaccines:</u> Antibody levels to specific vaccines as measured by ELISA.

<u>For immunogenicity to MVA.HIVA:</u> Frequency of IFN-γ-producing cells determined in an ELISPOT assay after overnight stimulation with a pool of HIVA-derived peptides. We shall consider the MVA.HIVA vaccine to be immunogenic in infants if at least 50% of vaccinees have detectable HIV-1-specific T cell responses at any time point after the vaccination. A positive response is defined to be at least 250 SFU/10^6 cells above the no-peptide background, and at least 4x the no-peptide background. We shall be using standardised

and trial-tested assays, which have been described in Goonetilleke et al. [60]. MVA.HIVA alone is not considered to be a strong priming vaccine, but it can strongly and consistently boost HIV-1-specific T cell responses if they have been previously primed [20, 21, 59, 60]. Thus, in future, the HIV-specific T cell levels after the MVA.HIVA vaccination are expected to be enhanced by the BCG.HIVA priming should the BCG.HIVA vaccine prove to be immunogenic in human infants.

The history of viral infections can influence the immune response to subsequent infections in mice (Selin, Immun Rev 2006). Preliminary evidence suggests the order and combination of vaccines also influences the type of vaccine induced immunity generated in children (Rowland-Jones, unpublished). We plan to measure humoral IgG immune responses to KEPI vaccines by commercial ELISA tests. There is little data available on immunologic cellular phenotypic changes that occur in infants receiving immunizations during their first year of life. We propose to evaluate changes in activation, maturation (CD45RO, RA, CCR7, CD69, CD27, CD28), and function (granzymes, CD107a, NK markers, Tregs) in CD4+ and CD8+ T cells.

Innate immunity is the link between antigen exposure and the adaptive immune response, and may influence the outcome of immunizations. Plasma levels of cytokines associated with activation of innate immunity will be measured by cytometric bead array assays.

For Capacity Building: One centre in Kenya will be ready for conducting infant HIV-1 vaccine clinical trials.

4 STUDY DESIGN

This is an open randomized phase I/II study evaluating the safety and immunogenicity of a single dose of MVA.HIVA vaccine, and its impact on the immunogenicity of administered KEPI vaccines in HIV-1 negative infants born to HIV-1-infected mothers. The immunology laboratory will be blinded for the study product/control group allocation.

Study site: Kenyatta National Hospital, Nairobi, Kenya

4.1 Sample size:

A total of 72 mother-infant pairs will be block randomised to receive either MVA.HIVA and KEPI vaccines or KEPI vaccines only. When infants are enrolled into the study they will be placed into either the breastfeeding (BF) cohort or the formula-feeding (FF) cohort, based on the preference of the mother after completing feeding counseling. An estimated 150 infants will be initially enrolled in this study to yield 72 infants eligible for randomisation.

Breastfeeding Cohort (BF): Safety and immunogenicity of MVA.HIVA administered to infants compared to age-matched unvaccinated controls. Two groups of 18 breastfed infants each born to HIV-1-infected mothers will be treated as follows:

Group 1: MVA.HIVA administered at week 20 (n=18)
Group 2 (Control Group): No MVA.HIVA (n=18)

Formula-feeding Cohort (FF): Safety and immunogenicity of MVA.HIVA administered to infants compared to age-matched unvaccinated controls. Two groups of 18 formula-fed infants each born to HIV-1-infected mothers will be treated as follows:

Group 3: MVA.HIVA administered at week 20 (n=18)
Group 4 (Control Group): No MVA.HIVA (n=18)

This study is a pilot study and the immunogenicity analyses will be mainly descriptive. As such, it is not powered to provide definitive evidence of vaccine-induced effects; rather it is based on smaller numbers of participants and will only indicate trends, feasibility, or size of effect and variability of measurements to inform the design of more definitive studies. The rate of severe and very severe adverse events will be used as one measure of the safety of the candidate vaccine. Table 2 shows examples of sample size calculations for safety in terms of the ability to detect SAEs, i.e., the probabilities of observing 0, 1+ (1 or more), 2+ or 3+ SAEs among a group of n=18 for a range of possible true event rates. If none of the 18 volunteers receiving the vaccine experiences an SAE definitely related to the vaccine, the 95% two-sided upper confidence bound for the rate of such reactions in the population is 18.5% (Table 3).

Table 2. Probabilities of detecting events for n=18

Event rate (SAE)	Probability (0/18 Events)	Probability (1+/18 events)	Probability (2+/18 events)	Probability (3+/18 events)
0.010	0.83	0.17	0.01	0.0007
0.025	0.63	0.37	0.07	0.0096
0.035	0.53	0.47	0.13	0.02
0.050	0.40	0.60	0.23	0.06
0.100	0.15	0.85	0.55	0.27
0.150	0.05	0.95	0.78	0.52
0.200	0.02	0.98	0.90	0.73
0.250	0.01	0.99	0.96	0.86

Table 3. 95% CI for the probability of SAE

Observed proportion of SAE	95% Confidence Interval
0/18	0-18.5%
1/18	0.1-27.3%
2/18	1.4-34.7%
3/18	3.6-41.4%
4/18	6.4-47.6%
5/18	9.7-53.5%

One of the outcomes of the study is determination of MVA.HIVA gross interference with the immunogenicity of KEPI vaccines. The trial will not be powered to detect small interference effects.

4.2 Randomization:

HIV-1 infected mothers attending one of the study recruitment antenatal clinics either at KNH or selected City Council of Nairobi clinics will be eligible for having their infants enrolled if they meet inclusion criteria and the study is still in an open enrolment phase. Women will be counseled on breast or formula feeding and will be supported in their feeding decision. Formula will be provided free of charge for women electing to formula feed. All mothers will receive HAART for prevention of mother-to-child transmission (PMTCT) of HIV-1 during pregnancy and delivery. Those mothers who choose to breastfeed will also receive HAART during the duration of breastfeeding and will be advised to wean when the child is 6 months old.

Mothers will enroll infants into the study during pregnancy through written informed consent, pending the infant meeting inclusion criteria at birth. At the age of 20 weeks, infants who meet all inclusion and none of the exclusion criteria will be randomized to one of the two study groups of 18 for each feeding cohort (Table 4). Randomization will occur at the time of study product vaccination (20 weeks). For each feeding cohort, randomization will be performed to allocate infants to receive either MVA.HIVA and KEPI (Groups 1 and 3) or KEPI alone (Groups 2 and 4). Randomisation will occur by picking the next envelope (block randomisation) assigning them to one of the treatment groups. Infants recruited into this study will receive their routine KEPI and study vaccines from the study team in order to reduce confounding variables that might result from variations in age at vaccination and

batch of vaccines administered.

Table 4. Study groups

Group	N	Feeding Arm	Treatment
1	18	Breastfeeding	$5x10^7$ pfu of MVA.HIVA immunisation and KEPI
2	18	Breastfeeding	KEPI alone (Control)
3	18	Formula-feeding	$5x10^7$ pfu of MVA.HIVA immunisation and KEPI
4	18	Formula-feeding	KEPI alone (Control)

Masking:
It will not be possible to have complete masking in this study. However, the personnel who are performing the immunology assays (primary outcome measures) will be masked as they will be dealing with coded laboratory specimens and will thus not know whether the specimen is from a subject who received the study vaccine or not.

5 STUDY POPULATION

Women presenting for antenatal care at Kenyatta National Hospital (KNH) or one of the identified antenatal care clinics located in the Nairobi area are offered HIV counseling and testing by a trained nurse/counselor through established PMTCT programs. During active enrolment for the study, if a woman in her second or third trimester is HIV-1-infected, she will be provided with introductory information about this vaccine study and be asked to return to KNH for a screening visit. The initial screening will include reviewing and signing the informed consent form, confirmatory HIV-1 testing by ELISA, determination of CD4 count, and a physical examination. Women with CD4 counts <350 cells/µL or WHO HIV stage 3 and 4 disease progression will not be eligible for this phase I/II study. As included in the most recent Kenyan national PMTCT guidelines, the HIV-1 infected women in this study will be offered HAART and the HIV-1 un-infected infants will receive a short course of ARVs after birth through the first 6 weeks of life. All study participants will be asked to register as antenatal clinic patients with KNH to receive an antiretroviral (ARV) regimen for PMTCT. After delivery each breastfeeding mother's health and response to her HAART regimen will be monitored at the post pregnancy care clinic and then the Comprehensive Care Clinic (CCC) at KNH, where she will receive her ARV medication during breastfeeding. The Kenyan Ministry of Health recommends that those women who are unable to safely formula feed their infants practice exclusive breastfeeding followed by weaning at 6 months of age for those who can safely do so. All breastfeeding women will be counseled to wean by the time their infants are 6 months old, but they will continue to receive ARVs if breastfeeding continues past that age.

All subjects must meet all of the inclusion criteria to participate in this study.

5.1 Inclusion Criteria

Mother Inclusion Criteria

a. Second or third trimester of pregnancy, as determined by a clinical exam and reported menstrual history
b. Written informed consent
c. ≥ 18 years of age
d. Confirmation of HIV-1 infection documented by ELISA
e. CD4 count > 350 cells/ µl in the screening blood specimen and at 6 weeks after delivery
f. Stated willingness to receive HAART during pregnancy and breastfeeding (if applicable)
g. Stated intent to deliver at Kenyatta National Hospital

h. Stated intent to remain in the Nairobi area for at least a year after delivery

Infant Inclusion Criteria

a. Healthy infants
b. $\leq$ 3 days of age (day of birth = Day 0) at enrolment
c. Birth weight $\geq$ 2500 grams
d. Born to an eligible woman
e. Written informed consent by parent

Table 5. Overview of Kenyan Expanded Programme on Immunization (KEPI) vaccination schedule in the first 12 months of life

Age	Vaccine Names	Additional Vaccine Descriptions
Birth	BCG, OPV birth	Bacillus Calmette-Guérin vaccine (against tuberculosis); Oral polio vaccine
6 weeks	DTP1, HiB1, HBV1, OPV1	Diphtheria, tetnus, and pertussis vaccine; Haemophilus influenzae type B vaccine (against meningitis); Hepatitis B virus vaccine; Oral polio vaccine
10 weeks	DTP2, HiB2, HBV2, OPV2	
14 weeks	DTP3, HiB3, HBV3, OPV3	
9 months	Measles	

5.2 Exclusion Criteria

Mother Exclusion Criteria

a. WHO stage 3 or 4 disease progression as determined by clinical evaulation
b. Prior participation in any HIV-1 vaccine or drug trial
c. Receipt of any investigational agent during this pregnancy
d. Receipt of blood products, immunoglobulin, or immunotherapy during this pregnancy
e. Evidence of clinically significant disease that would compromise the ability of the participant to complete the study or the study requirements as determined by the study clinician
f. Known multiple gestation in the current pregnancy

Infant Exclusion Criteria

a. HIV infection, as determined by a filter paper and/or RNA test prior to vaccination.
b. Participation in any other HIV-1 vaccine or drug trial.
c. Failure to receive all standard KEPI immunizations according to national immunization programme (Table 5).
d. Weight for age z-scores outside of 2 standard deviations of normal at the time of vaccination.
e. Acute disease at the time of vaccination (acute disease is defined as the presence of a moderate or severe illness with or without fever). All vaccines can be administered to persons with a minor illness such as diarrhoea, mild upper respiratory tract infection with or without low-grade febrile illness, i.e., temperature of <37.5 °C).
f. Axillary temperature of ≥ 37.5 °C at the time of vaccination.
g. Any clinically significant abnormal finding on screening from biochemistry or haematology by the time of vaccination.
h. History of allergic disease or reactions likely to be exacerbated by any component of the vaccine, e.g., egg products.
i. Presence of any underlying disease that compromises the diagnosis and evaluation of response to the vaccine.
j. Any other on-going chronic illness requiring hospital specialist supervision.

k. Administration of immunoglobulins and/or any blood products within one month preceding the planned administration of the vaccine candidate.
l. Any history of anaphylaxis in reaction to vaccination.
m. Research Physician's assessment of lack of willingness by parents to participate and comply with all requirements of the protocol, or identification of any factor felt to significantly increase the infant's risk of suffering an adverse outcome.
n. Likelihood of travel away from the study area.

5.3 Sensitisation and Recruitment Procedures

During the recruitment phase of the study, parents of infants attending one of the antenatal recruitment clinics will be approached to have their children enrolled in this study. Both mothers and fathers will be involved in the recruitment and infant consent process. The purpose of the study will be described to the parents of prospective study infants and what it will involve for the infants, including the study screening and the informed consent procedure, methods of vaccination, observation details, spectrum of likely side effects/adverse events, follow-up details and extent of blood sampling, as well as risks of vaccination and the unproven benefits of vaccination. The need for an HIV-1 vaccine, including a simple picture of the burden of HIV-1 on the community, country and Africa and the current status of HIV-1 vaccine development, including the fact that it is likely to be a prolonged process, will be explained. It will be stressed that this is an experimental vaccine and it cannot be guaranteed to provide protection. The need for a health screening before receiving vaccinations because the vaccine is in an early stage of development will be explained. In addition they will be provided with the trial's information sheet for their use and to show to their spouses or relatives. All information provided to parents will be submitted to the local Ethics and Research Committee for approval. All information will be available in both English and Kiswahili.

Parents must understand that these vaccines cannot cause HIV or AIDS. Parents must understand that the MVA.HIVA vaccine has not yet been shown to prevent HIV-1 infection and this will be stressed during the recruitment stage. Infants should not be subjects in any other vaccine trial or any drug trial. It will be stressed that the infants can be withdrawn from the study at any stage.

If parents are interested in participating in the study, they will be asked to review the clinical trial information sheet and come to the KNH clinic for a screening visit prior to labour and delivery. It will be explained that this screening visit should occur before or as close to 28 weeks gestation as possible to start the mother on the appropriate PMTCT drug regimen. At this screening visit, the subject's parents will have the study explained again, be asked to read the informed consent form, and will undergo an assessment for their understanding of the study. If the parents have no further questions and pass the understanding assessment, they will be asked to sign (or thumb-print) an informed consent document, which is approved by the Kenyatta National Hospital/University of Nairobi Ethics and Research Committee, the University of Washington Institutional Review Board, and Oxford Tropical Research Ethics Committee (OXTREC) prior to any study-related evaluations being performed (see informed consent form, Appendix 2). One copy of the signed consent form will be given to the parents and a second copy will be retained by the investigators for the infants' study records. Those infants whose parents have signed consent documentation will be assessed at Kenyatta National Hospital as described below in the study schedule.

The study will be conducted in accordance with the International Conference on Harmonization Good Clinical Practice (ICH-GCP) guidelines.

6 STUDY ENROLMENT AND SCHEDULE OF FOLLOW-UP VISITS

This trial includes a screening of women who meet all the inclusion criteria and none of the exclusion criteria. Each eligible woman will need to indicate whether she plans on breastfeeding or formula-feeding her infant and will receive appropriate feeding counselling

to help her with her decision. Written informed consent at the screening visit prior to delivery must be obtained before the following procedures can take place.

- Targeted medical history (brief medical, obstetric, HIV-1)
- Targeted physical examination (for gestational age, multiple pregnancy, presence of serious illness)
- Haematology (CBC with differential, CD4 count)
- HIV-1 ELISA or PCR (for HIV-1 status confirmation)
- Quantitative HIV-1 RNA PCR (stored plasma for later assay, if infant enrolled)
- HLA typing
- Dried blood spot, plasma, and PBMC for storage
- Assessment of understanding (prior to signing consent for infant enrolment)

Mothers' CD4 counts will be measured twice, once at the initial screening during the second or third trimester and once 6 weeks after delivery at study visit number 2, when a mother's CD4 count will be more physiologically stable than during pregnancy and delivery.

During the screening visit, the study physician will make sure that the mother understands the risks and benefits of receiving HAART during pregnancy and breastfeeding (if applicable) for PMTCT. The mother will receive referral to the antenatal clinic at KNH for the administration and monitoring of HAART during pregnancy. After delivery she will be followed at a post pregnancy care clinic. Mothers who choose to breastfeed their infants will continue to receive HAART after delivery from the CCC. The PMTCT regimen includes three types of ARV drugs taken twice daily from 28 weeks of pregnancy to delivery for all mothers, to be continued until weaning for those mothers who breastfeed their infants. The recommended dosing for the mothers includes azidothymidine (AZT) 300 mg bi-daily (BD), lamivudine (3TC) 150mg BD, and a combination of lopinavir (LPV) 400 mg and ritonavir 100 mg BD. All infants will receive nevirapine (NVP) 2 mg per kilogram of body weight (mg/kg) within 72 hours of birth, 3TC 2 mg/kg daily for 1 week, and AZT 4 mg/kg daily for 6 weeks.

Infants who meet all inclusion and none of the exclusion criteria will be recruited and placed into either the BF or FF cohort, according to the parents' feeding decisions. If the parents change their mind about the feeding method during the months leading up to the randomisation day or if responses to feeding practice questionnaires suggest that the infant is not being fed according to the parent's initial BF or FF choice, the infant's case will be evaluated to determine if it is appropriate to switch the subject from one study cohort to the other and if it is appropriate to change the mother's PMTCT regimen to reflect the infant's new exposure level to HIV. At 20 weeks of age, infants will be consecutively randomized to one of the two groups of 18 (Table 4).

Infants recruited into this study will receive their routine KEPI and study vaccines from the study team in order to reduce confounding variables that might result from variations in age at vaccination, and batch of vaccines administered.

A sticker indicating that the child is involved in a study and should only be given KEPI vaccines by the study team up to the age of 9 months will be placed on the health card. This sticker will not identify the name of the study or any identifying information that might unintentionally disclose the mother's HIV status.

Table 6 illustrates the sequence of events from recruitment, through MVA.HIVA vaccination and subsequent follow-up visits (explained in more detail in the text that follows). Table 7 outlines the timing of infant and mother blood investigations.

Table 6. Illustration of sequential events from recruitment through vaccination, & and subsequent follow-up visits.

Study Visit	Age of Infant	Schedule relative to study product administration	Mother Procedures	Infant Procedures	
				All Infants (Groups 1-4)	Groups 1 and 3 Only
Recruitment	2nd or 3rd Trimester	n/a	Introduction to study	n/a	
Screening	2nd or 3rd Trimester	n/a	Informed consent; physical exam; feeding counseling; **blood draw**	n/a	
Enrolment	Birth	-20 weeks	Delivery; continued consent; feeding counseling	Physical exam; subject ID given; **heel prick**; KEPI	
1	2 weeks	-16 weeks	Continued consent; feeding counseling	Physical exam	
2	6 weeks	-14 weeks	Continued consent; feeding counseling; **blood draw**	Physical exam; **heel prick**; KEPI	
3	10 weeks	-10 weeks	Continued consent; feeding counseling	Physical exam; **heel prick**; KEPI	
4	14 weeks	-6 weeks	Continued consent; feeding counseling	Physical exam; **heel prick**; KEPI	
5	19 weeks	-1 week	Continued consent; feeding counseling	Physical exam; **blood draw**	
6	20 weeks	0 weeks	Continued consent; feeding counseling	Physical exam; **randomisation**; **heel prick**	**MVA.HIVA**
Home visit 1	20 weeks, 1 day	+1 day	Continued consent	n/a	Vital signs; injection site checked
Home visit 2	20 weeks, 2 days	+2 days	Continued consent	n/a	Vital signs; injection site checked
7	21 weeks	+1 week	Continued consent; feeding/weaning counseling	Physical exam; **blood draw**	Injection site checked
8	28 weeks	+8 weeks	Continued consent; feeding/weaning counseling	Physical exam; **blood draw**	Injection site checked
9	36 weeks	+16 weeks	Continued consent; feeding/weaning counseling	Physical exam; **blood draw**; KEPI	Injection site checked
10	48 weeks	+28 weeks	Continued consent; feeding/weaning counseling; **ESP**	Physical exam; **blood draw**; **ESP**	Injection site checked

Blood draw = 5 ml of blood; **heel prick** = drops of blood from heel; KEPI = Vaccines of the Kenyan Expanded Programme on Immunization; **Randomisation** = Assignment into Group 1, 2, 3, or 4; **MVA.HIVA** = HIV-1 vaccine candidate; **ESP** = End of study protocol

Table 7. Temporal course of exact subject blood investigations

Investigation	Screen-ing Mother	Birth Infant	Wk 6 Mother	Wk 6 Infant	Wk 10 Infant	Wk 14 Infant	Wk 19 Infant	Wk20 Infant	Wk21 Infant	Wk 28 Infant	Wk 36 (9 mo) Infant	Wk 48 (12mo) Infant
Haematology	X						X		X	X		
Biochemistry							X		X	X		
MVA.HIVA Immunogenicity							X		X	X	X	X
KEPI Immunogenicity							X		X			
CD4 Count	X		X				X			X	X	X
HIV-1 test	X	X		X	X	X	X	X		X	X	X
HLA typing (if necessary)	X											X
Specimen Archive	X		X				X		X	X	X	X

6.1 Definitions

Haematology Full blood count (FBC) including platelet count
Biochemistry Creatinine and alanine transaminase (ALT)
MVA.HIVA immunogenicity Frequency of IFN-γ producing cells using ELISPOT assays after overnight stimulation with pool(s) of 15-mer peptides overlapping by 11 amino acids spanning the length of the gag region and a pool corresponding to the polyepitope region of of immunogen HIVA. The MVA.HIVA vaccine will be considered immunogenic in infants if at least 50% of vaccinees have detectable HIV-1-specific T cell responses at any time point after the vaccination.
KEPI immunogenicity Measurement of vaccine-induced antibody levels to DTP, HiB, HBV and OPV.

Dissemination and explanation of blood results

All non-immunology blood results will be given and explained to all parents after screening and at follow-up visits. If any infant is found to have antibody titres to KEPI vaccines below the level of protection, this immunology result will be given and explained to that infant's parent and the infant will receive a booster dose of the vaccine in question. All other immunology results will be explained in general terms, for the group and not for individuals, at the end of the study. Those with abnormal blood results at screening or during the course of the study will be offered appropriate investigations and treatment or referral as necessary. Parents will receive an explanation of all screening results before proceeding with MVA.HIVA immunization.

6.2 Study Visits:

At Delivery (enrolment visit): During or after labour, mothers and fathers will be asked to confirm their continued consent to participate in the study. The newborn will be examined for abnormalities and drops of blood will be collected from the infant's heel for an HIV-1 filter paper and/or RNA test. If the inclusion criteria are fulfilled and none of the exclusion criteria are met, a study number will be assigned to the subject. This study number will be used to track all visits and clinical and laboratory data for the duration of the infant's involvement in the study. The KEPI vaccines due at birth (BCG and OPV) will be given prior to discharge. All mothers will receive feeding counseling and each mother's feeding choice will be confirmed. Infants in the FF cohort will receive free formula at this visit. At delivery, the post pregnancy care clinic will ensure that the mother and infant receive the appropriate antiretroviral regimen to prevent mother to child transmission, which includes NVP 2 mg/kg

within 72 hours of delivery for all infants. The mother should already be taking HAART, but if she has not been following her prescribed drug regimen, she will receive NVP 200 mg right away and AZT 300 mg BD and 3TC 150 mg BD for one week. An appointment will be arranged for the mother to come to the clinic for the first post-natal visit at 2 weeks of age for a follow-up feeding counseling visit to support her feeding choice.

Visit 1 (Infant aged 2 weeks, ± 1 week)

- Confirmation of continued consent to participate in the study.
- An interim medical history will be taken.
- Physical examination performed by the Research Physician, including vital signs (temperature, respiratory rate and pulse rate), weight, length and mid-left upper arm circumference (MUAC), will be documented.
- Study staff will have a follow-up feeding counseling session with the mother, confirming and supporting her feeding choice.
- Formula feed provided to those who have chosen to formula feed.
- Appointment given for 4 weeks time.

Visit 2 (Infant aged 6 weeks, ± 1 week)

- Confirmation of continued consent to participate in the study.
- An interim medical history will be taken.
- Physical examination performed by the Research Physician, including vital signs (temperature, respiratory rate and pulse rate), weight, length and mid-left upper arm circumference (MUAC), will be documented.
- Administration of appropriate KEPI vaccines for age (DTP1, HiB1, HBV1, and OPV1).
- Drops of blood collected from infant's heel for HIV-1 filter paper and/or RNA test.
- 5 ml of venous blood collected from the mother for a second CD4 test for screening purposes.
- Study staff will record subjects' feeding practices in the case report form (CRF) and will confirm and support the mother's feeding choice.
- Formula feed provided to those who have chosen to formula feed.
- Appointment given for 4 weeks time.

.

Visit 3 (Infant aged 10 weeks, ± 1 week)

- Continued consent confirmed.
- An interim history will be taken.
- Physical examination performed by the Research Physician, including vital signs (temperature, respiratory and pulse rates), weight, length and MUAC, will be documented.
- KEPI vaccines appropriate for age and study group will be administered (DTP2, HiB2, HBV2, and OPV2).
- Drops of blood collected from infant's heel for HIV-1 filter paper and/or RNA test.
- Study staff will record subjects' feeding practices in the case report form (CRF) and will confirm and support the mother's feeding choice.
- Formula feed provided to those who have chosen to formula feed.
- Appointment given for 4 weeks time.

Visit 4 (Infant aged 14 ± 1 weeks)

- Subjects will be brought to the vaccine trial clinic.
- Continued consent confirmed.
- An interim history will be taken.
- Physical examination performed by the Research Physician, including vital signs (temperature, respiratory and pulse rates), weight, length and MUAC, will be documented.
- KEPI vaccines appropriate for age and study group will be given (DTP3, HiB3, HBV3, and OPV3).

- Drops of blood collected from infant's heel for HIV-1 filter paper and/or RNA test.
- Study staff will record subjects' feeding practices in the case report form (CRF) and will confirm and support the mother's feeding choice.
- Formula feed provided to those who have chosen to formula feed.
- Appointment given for 5 weeks time.

Visit 5 (Infant aged 19 weeks, ± 1 week)

- Subjects will be brought to the vaccine trial clinic.
- Continued consent confirmed.
- An interim history will be taken.
- Physical examination performed by the Research Physician, including vital signs (temperature, respiratory and pulse rates), weight, length and MUAC, will be documented.
- 5 ml of venous blood collected for baseline haematology, biochemistry, HIV-1 filter paper and/or RNA test, and pre-MVA.HIVA immunogenicity and KEPI vaccine antibody assays. If this blood draw collects less than 3 ml of venous blood, a note will be left in the infant's file for an additional 3 ml of venous blood to be collected at the next visit and before any study vaccination.
- Study staff will record subjects' feeding practices in the case report form (CRF) and will confirm and support the mother's feeding choice.
- Formula feed provided to those who have chosen to formula feed.
- Appointment at the clinic given for 1 week's time.

Visit 6, MVA.HIVA vaccination day (Infant aged 20 weeks, ± 1 week)

- Continued consent confirmed.
- An interim history will be taken.
- Inclusion and exclusion criteria will be checked.
- Results of the haemotology and biochemistry laboratory tests from the previous week's blood draw will be reviewed by the research physician to ensure the child is eligible for randomisation.
- Physical examination performed by the Research Physician, including vital signs (temperature, respiratory and pulse rates), weight, length and MUAC, will be documented.
- Drops of blood collected from infant's heel for HIV-1 filter paper and/or RNA test. If previous clinic visit's blood draw collected less than 3 ml of venous blood, 3 ml of venous blood will be collected to complete the baseline laboratory tests. In this case, no heel prick is necessary.
- Study staff will record subjects' feeding practices in the case report form (CRF) and will confirm and support the mother's feeding choice.
- Formula feed provided to those who have chosen to formula feed.
- The screening safety profile will be reviewed and those infants that fulfil all inclusion criteria and no exclusion criteria will be randomised into treatment groups. If any infant fails to meet the contraindications to MVA.HIVA administration included in the exclusion criteria at the time scheduled for vaccination, the subject may be vaccinated at a later date when those criteria are met (within a week), or withdrawn at the discretion of the investigator. Infants will be randomised to a group (Group 1-4) by the trial physician by selecting an envelope from those prepared for the breastfeeding or formula feeding group, as appropriate.
- The Groups 1 and 3 infants will receive the MVA.HIVA vaccine (5×10^7 pfu), administered intramuscularly into the deltoid muscle of the left arm with a BCG scar. The injection site will be covered after 10 minutes with a sterile dressing that will be removed at the one-hour observation.
- The infant and parent will remain at the clinical area for one hour following vaccination and will then be allowed to return home.

- Vital signs will be checked at 30 minutes (+/- 5 min) and 1 hour (+/-10 min) post injection.
- The injection site will be inspected at 1 hour (+/-10 min) and the largest diameter of any induration or redness will be documented into the CRF. An assessment for possible systemic AE's will be carried out and any concomitant medication documented.
- Appointment given for 1 week's time.
- Nurse/field worker will visit subjects at home daily for the first two days after MVA.HIVA vaccination to record safety and reactogenicity data on a standard CRF. This staff member will not wear a uniform or other identifying clothing and will take precautions against any unintended disclosure of a woman's HIV status or participation in this study.

Visit 7, Post-vaccination week 1 (Infant aged 21 ± 1 week)

- Subjects will be brought to the vaccine trial clinic.
- Continued consent confirmed.
- An interim history will be taken.
- Physical examination performed by the Research Physician, including vital signs (temperature, respiratory and pulse rates), weight, length and MUAC, will be documented.
- The MVA.HIVA injection site will be inspected and the largest diameter of any induration or redness present will be documented into the CRF.
- Solicited adverse events (local and systemic) that may have occurred since the infant was vaccinated and any concomitant medications will be documented in the CRF.
- 5 ml of blood will be collected for haematology, biochemistry, immunogenicity of MVA.HIVA, and KEPI vaccine antibody assays.
- Study staff will record subjects' feeding practices in the case report form (CRF), will confirm and support the mother's feeding choice, and will counsel about appropriate weaning practices.
- Formula feed provided to those who have chosen to formula feed.
- Appointment card given for 7 weeks' time.

Visit 8 (Infant aged 28 weeks, ± 2 weeks)

- Subjects will be brought to the vaccine trial clinic.
- Continued consent confirmed.
- An interim history will be taken.
- Physical examination performed by the Research Physician, including vital signs (temperature, respiratory and pulse rates), weight, length and MUAC, will be documented.
- For infants in Groups 1 and 3, the MVA.HIVA injection site will be inspected and findings documented in the CRF.
- Solicited adverse events (local and systemic) that may have occurred since the last visit and any concomitant medications will be documented in the CRF.
- 5 ml of blood will be collected for haematology, biochemistry, HIV-1 testing, CD4 count and immunogenicity of MVA.HIVA.
- Study staff will record subjects' feeding practices in the case report form (CRF), will confirm and support the mother's feeding choice, and will counsel about appropriate weaning practices.
- Formula feed provided to those who have chosen to formula feed.
- Appointment at the clinic given for when the infant is 36 weeks old (9 months).

Visit 9 (Infant aged 36 weeks, ± 2 weeks)

- Subjects will be brought to the vaccine trial clinic.
- Continued consent confirmed.
- An interim history will be taken.

- Physical examination performed by the Research Physician, including vital signs (temperature, respiratory and pulse rates), weight, length and MUAC, will be documented.
- For infants in Groups 1 and 3, the MVA.HIVA injection site will be inspected and findings documented in the CRF.
- Solicited adverse events (local and systemic) that may have occurred since the last visit and any concomitant medications will be documented in the CRF.
- KEPI vaccines appropriate for age and study group will be given (measles)
- 5 ml of blood will be collected for HIV-1 testing, CD4 count and immunogenicity of MVA.HIVA.
- Study staff will record subjects' feeding practices in the case report form (CRF), will confirm and support the mother's feeding choice, and will counsel about appropriate weaning practices.
- Formula feed provided to those who have chosen to formula feed.
- Appointment at the clinic given for when the infant is 48 weeks old.

Visit 10 (Infant aged 48 weeks, ± 2 weeks)

- Subjects will be brought to the vaccine trial clinic.
- An interim history will be taken.
- Physical examination performed by the Research Physician, including vital signs (temperature, respiratory and pulse rates), weight, length and MUAC, will be documented.
- For infants in Groups 1 and 3, the MVA.HIVA injection site will be inspected and findings documented in the CRF.
- Solicited adverse events (local and general) that may have occurred since the last visit and any concomitant medications will be documented in the CRF.
- 5 ml of blood will be collected for HIV-1 testing, CD4 count and immunogenicity of MVA.HIVA.
- Study staff will record subjects' feeding practices in the case report form (CRF), will confirm and support the mother's feeding choice, and will counsel about appropriate weaning practices.

Parents will be told that their babies have graduated from the study. The study sticker will be removed from their health cards.

7 STUDY INTERVENTION / INVESTIGATIONAL PRODUCT

Study Product Acquisition

Study product will be shipped by IDT Biologika GmbH, am Pharmapark, D-06861 Dessau-Rosslau, Germany, directly to the trial site.

Formulation, Packaging and Labelling

MVA.HIVA is manufactured according to Good Manufacturing Practice (GMP) conditions by Impfstoffwerk Dessau-Tornau (IDT), Germany, as contracted by Medical Research Council. It is supplied as a ready-to-use solution in glass vials. These vials are clearly labelled as containing MVA.HIVA in accordance with GMP guidelines on investigational medicinal products.

Product Storage and Stability

MVA.HIVA will be stored at –20 °C in a locked freezer at the trial clinic laboratory.

Preparation, Administration and Dosage of Study Intervention/Investigational Product

The batch (number to be determined) of the vaccine, MVA.HIVA, will be provided in vials of 200 µl at a concentration of no less than $5x10^8$ pfu/ml in 10 mM Tris buffer, 0.8% NaCl. The dose of MVA.HIVA to be used in this study will be $5x10^7$ pfu.

On the vaccination day, vaccines will be allowed to thaw to room temperature and

administered within 60 minutes. One vaccine vial per subject to be vaccinated will be used. The vaccine will be administered intramuscularly over the deltoid region of the left arm (the arm usually displaying a BCG scar). The investigator will wear gloves and eye protection. Subjects will stay in the health centre for observation for at least 60 minutes after vaccination. During administration of the vaccines, medicines and resuscitation equipment will be immediately available for the management of anaphylaxis.

In order to minimise dissemination of the recombinant vectored vaccine virus into the environment the inoculation site will be covered with a dressing 10 minutes after immunization. This should absorb any virus that may leak out through the needle track. The dressing will be removed from the injection site at the end of the 60-minute observation period and will be disposed as GMO waste by autoclaving and incineration, in accordance with the relevant SOP and current standard Kenyan practice.

8 ASSESSMENT OF SCIENTIFIC OBJECTIVES

Specification of the Appropriate Outcome Measures

Primary Outcome Measures:

- The safety of MVA.HIVA will be determined by analysis of adverse events, reactogenicity, biochemical and haematological data that will be actively collected.

- Assessment of the immunogenicity of a single dose of MVA.HIVA administered separately from KEPI vaccines in healthy infants who have been vaccinated with BCG will be by measuring the frequency of IFN-γ-producing cells following overnight stimulation with pooled 15-mer peptides derived from the HIVA immunogen in ELISPOT assays.

Secondary Outcome Measures:

- Determination of gross interference of MVA.HIVA with the immunogenicity of DTP, HiB, HBV and OPV vaccines will be through comparing actively collected data on antibody levels to the antigens contained in these KEPI vaccines in the vaccine and control groups. Historical data from other studies in the same population may also be used to provide normal ranges.

- If there are any residual PBMCs after the IFN-γ ELISPOT assays have been performed, other immunological assays may be performed, which might include:

  - Cultured IFN-γ ELISPOT assays for non-vaccine HIV-1 peptides
  - *Ex vivo* ELISPOT assays for IFN-γ, interleukin 2 or tumour necrosis factor alpha, following stimulation with HIVA and HIV-1 peptides
  - T cell proliferation to HIVA and HIV-1 peptides
  - T cell depletion assays
  - Cytokine detection using ELISA/LUMINEX or
  - Intracellular cytokine staining and multicolour flow cytometric analysis

9 ASSESSMENT OF SAFETY

All adverse events (AE) occurring in participants after administration of vaccine will be documented and reported as described below.

9.1 Definitions

Adverse Event

An AE is any untoward medical occurrence, including a dosing error, which may occur during or after vaccination. The AE may or may not have a causal relationship with vaccination as indicated by physical signs, symptoms, and/or clinically significant laboratory abnormalities that occur. The definition includes intercurrent illnesses, injuries, exacerbation of pre-existing conditions, and events occurring as a result of product misuse or overdose.

A change in a laboratory variable is considered an AE if it leads to a change in the subject's functional status or if it is considered by the attending physician to be clinically significant. The following laboratory studies will be monitored during the course of this study (as previously outlined): FBC, creatinine, and ALT.

Expected local reactions to the vaccine will not be recorded as an adverse event, but will be recorded on the CRFs. These include:

- Redness
- Induration
- Scaling/Blistering/Ulceration
- Axillary lymph node enlargement
- Pain (scored as follows)
  - 0 No pain
  - 1 Painful to touch
  - 2 Partial restriction of activities
  - 3 Unable to use the arm

Serious Adverse Event (SAE)

Any AE (whether or not considered related to the investigational product) that is categorized as Grade 3 (severe) or Grade 4 (potentially life-threatening) according to the Division of AIDS Table for Grading the Severity of Adult and Paediatric Adverse Events (DAIDS AE grading table) is defined as an SAE. Any of the following outcomes will be considered an SAE:

1) Death (from any cause at any time).

2) Life-threatening event (i.e. the subject was, in the view of the investigator, at immediate risk of death from the event that occurred). This does not include an AE that, if it occurred in a more serious form, might have caused death.

3) Persistent or significant disability or incapacity (i.e., substantial disruption of one's ability to carry out normal life functions).

4) Hospitalisation: regardless of length of stay, even if the hospitalization is a precautionary measure for continued observation. Hospitalisation (including inpatient or outpatient hospitalization for an elective procedure) for a pre-existing condition that has not worsened unexpectedly does not constitute a serious AE.

5) An important medical event (that may not cause death, be life threatening, or require hospitalization) that may, based upon appropriate medical judgment, jeopardize the subject and/or require medical or surgical intervention to prevent one of the outcomes listed above. Examples of such medical events include allergic reaction requiring intensive treatment in an emergency room or clinic, blood dyscrasias, convulsions that do not result in inpatient hospitalization, or an infant's seroconversion to HIV-infected during the course of the study.

9.2 Adverse Events Assessment

For every adverse event (AE), an assessment of the relationship of the event to the

administration of the vaccine will be undertaken. An intervention-related AE refers to an AE for which there is a probable or definite relationship to administration of a vaccine. The investigator will make interpretation of the causal relationship of the intervention to the AE in question. This interpretation will be based on the type of event, the relationship of the event to the time of vaccine administration, and the known biology of the vaccine therapy. The following are guidelines for assessing the relationship of administration of one of the study vaccines to the AE.

1. NO RELATIONSHIP:
    - No temporal relationship to study product; _and_
    - Alternate aetiology (clinical state, environmental or other interventions); _and_
    - Does not follow known pattern of response to study product

2. POSSIBLE:
    - Reasonable temporal relationship to study product; _or_
    - Event not readily produced by clinical state, environmental or other interventions; _or_
    - Similar pattern of response to that seen with other vaccines

3. PROBABLE:
    - Reasonable temporal relationship to study product; _and_
    - Event not readily produced by clinical state, environment, or other interventions _or_
    - Known pattern of response seen with other vaccines

4. DEFINITE:
    - Reasonable temporal relationship to study product; _and_
    - Event not readily produced by clinical state, environment, or other interventions; _and_
    - Known pattern of response seen with other vaccines

Every AE observed or reported from the day of MVA.HIVA injection through to the end of the trial will be recorded on the appropriate CRF.

All AEs will be followed until resolution of the signs or symptoms or laboratory changes occurs, or until a non-study related causality is assigned.

Parents will be asked to assess the relationship of any adverse events to the vaccination (yes/no). These data will be used to aid the investigator, however a final decision on whether events are vaccine related or not is at the discretion of the investigator.

Serious Adverse Event (SAE) Reporting

In order to comply with current regulations on serious adverse event reporting the investigator pledges to document accurately the event, to respect notification deadlines, to provide the Sponsor with all necessary information and, if requested by the Sponsor, to give access to source documents.

All SAEs will be reported by telephone or in writing by email or fax to the Sponsor and Local Safety Monitor within one working day (24 hours) of the investigator becoming aware of the SAE occurrence.

Unexpected SAEs that are, in the opinion of the Principal Investigator or designated research physician, related to the administration of any of the research procedures (i.e. suspected unexpected serious adverse reactions (SUSARs)) should be reported to the local, UW and OXTREC Ethics Committees within 15 calendar days of the PI becoming aware of the event. If the event is fatal or life threatening, the event should be reported within 7 calendar days.

Minimum details to be given in a report are:

- Name of reporting doctor and contact telephone number.
- Study number.
- Nature of adverse event.
- Subject details (number sex, date of birth, weight and age).
- Date and time of event.
- Date and time of MVA.HIVA administration and dose.
- Other drug history.
- Other relevant history.
- Outcome.
- Causality.

The event will be documented on the SAE page of the CRF and reported to the Sponsor, Local Safety Monitor, Data Monitoring Ethics Committee (DMEC), relevant Ethics Committee, TSC, collaborators and funding agency as appropriate.

After the local ethics committee's response to the SAE is received, the Sponsor, Principal Investigator, Local Safety Monitor and available co-investigators will communicate to determine the future plan for the study, which could involve amending the protocol, discontinuing the vaccinations, or continuing unchanged for the other volunteers.

The study does not expect any SAEs to be related to the product vaccine for this study, but acknowledges that the study subjects are of an age when adverse health events are often seen in the population. As a result, there could be many AEs or SAEs documented during the course of the study; however, none are anticipated to be intervention-related.

A suspected unexpected serious adverse reaction (SUSAR) is different from other SAEs in that it is unexpected and thought to be related to the product vaccine for this study, MVA.HIVA. There are no expected serious adverse events associated with the vaccine. If an unexpected adverse reaction to the trial vaccine is suspected, the Sponsor will comply with the regulatory requirements on reporting SUSARs. The Sponsor pledges to inform the Authorities of any trial discontinuation and specify the reason for discontinuation.

9.3 Adverse Event Monitoring

Local Safety Monitor and Safety Committee

A Local Safety Monitor (LSM), Jacquelyn Nyange, will provide real-time safety oversight. The LSM will review SAEs immediately after they occur and follow these events until resolution. The LSM has the power to suspend enrolment into the study if deemed necessary following an SAE and convene a meeting with the Sponsor and Principal Investigator (PI) immediately. The LSM can convene a meeting with the Sponsor, PI, or other parties involved at any time for further discussion as necessary.

Methods and Timing for Assessing, Recording, and Analyzing Safety Parameters

AEs will be graded according to the DAIDS AE grading table, published in 2004. The DAIDS AE grading table classifies adverse events into one of four grades, ranging from mild to potentially life-threatening. The DAIDS AE grading table has indications for each of over sixty clinical parameters and forty laboratory parameters for grading adult and paediatric AEs. The table also includes general guidelines for estimating the grade of parameters not explicitly listed. Each grade is described broadly below:

- Grade 0: absence of the indicated symptom.
- Grade 1 (mild): awareness of a symptom, but the symptom is easily tolerated and causes no or minimal interference with usual activity.
- Grade 2 (moderate): discomfort enough to cause greater than minimal interference with usual activity.
- Grade 3 (severe): incapacitating; symptoms causing inability to perform usual activities; requires absenteeism or bed rest.

- Grade 4 (potentially life-threatening): symptoms causing inability to perform basic self-care functions OR medical or operative intervention is indicated to prevent permanent impairment, persistent disability or death.

Laboratory tests will also be graded on the DAIDS AE grading table.

Procedures to be Followed in the Event of Abnormal Laboratory Test Values or Abnormal Clinical Findings

Any infant found to have a clinically significant condition (e.g. heart failure, respiratory distress) prior to inclusion in the study or an abnormal biochemical or haematological result may have the test repeated to ensure this was not a laboratory error or spurious result. If the test remains clinically significant the infant will be excluded from the study and referred to their general physician as appropriate for further investigation and management with the permission of their parents. If occurring during the trial, tests may be repeated to verify the results, and further tests may be instigated to establish causality. If necessary the child will be withdrawn from the study. The infant's parents will be kept informed of all such occurrences and advised of actions to be taken, and their verbal/written consent taken for extra tests where appropriate. The trial physician will determine whether an abnormal laboratory or clinical finding would necessitate withdrawing the child from the study.

Halting Rules

Any SUSAR will result in suspension of the trial until a safety review is convened. The trial may be discontinued by the Kenyatta National Hospital/University of Nairobi Ethics and Research Committee, University of Washington Institutional Review Board, Oxford Tropical Research Ethics Committee (OXTREC) and Sponsor.

The Sponsor delegates to the DMEC the right to temporarily suspend or prematurely discontinue this trial at any time and for any reason as described in the DMEC charter. If the trial is stopped or suspended prematurely, the Sponsor will inform the Principal Investigator. If such action is taken, all effort must be made to ensure the safety of the participants enrolled in the study. The Principal Investigator will inform the relevant ethics committees about the decision and provide the reason for the suspension or termination.

10 CLINICAL MONITORING STRUCTURE

Site Monitoring Plan

Independent trial monitors will be contracted by the Medical Research Council and they will be responsible for the monitoring of this trial and compliance to ICH-GCP Guidelines. The Local Trial Monitor (LTM), Joyce Katuu, will be a valuable resource on-site and will work collaboratively with the Sponsor's monitor.

Pre-study Visit

A pre-study visit will be performed before the inclusion of the first infant in the study. This meeting will verify and document that the material to be used during the trial has been received and that the investigational team has been properly informed about the trial, regulatory requirements, and the SOPs. The pre-study visit will be coordinated between the local and sponsor monitors.

During this visit the Sponsor's monitor will run through the study flow with the trial team alongside all the documents and ensure everyone is confident in all aspect of the trial, how it will run and how the forms need to be completed. The monitors will conduct GCP refresher training if required and also explain the safety reporting system.

Follow-up Visits

The Sponsor's monitor will carry out regular follow-up visits. The Principal Investigator will be available for these visits and should allow the monitoring staff direct access to subjects'

medical file and CRFs. All trial monitors are committed to professional secrecy.
During the visits, the sponsor's monitor will:

- carry out a quality control of trial progress: respect of protocol and operating guidelines, data collection, signature of consent forms, completion of documents and appearance of SAEs,
- sample and product management, cold chain monitoring,
- collect the CRFs and corresponding correction sheets,
- evaluate the number of complete or on-going study participation events.

The trial monitors shall discuss any problem with the Principal Investigator and define with him the actions to be taken.

Once the CRFs corresponding to the last visit have been returned duly completed and signed, the investigator must be available to deal with any issues raised by the trial monitor, if necessary, until the database is locked.

In addition to the Sponsor's trial monitor, the local monitor will also carry out regular meetings with the trial staff, where the documents and compliance to GCP will be checked and feedback including advice and training recommendations will be given to the Principal Investigator.

The Sponsor's monitor will coordinate his/her visits with the local monitor and they will share their reports.

Close-out Visit

A close-out visit will be performed at the end of the trial by the Sponsor's and local trial monitors. Its goals are to make sure that:

- The centre has all the documents necessary for archiving.
- All paperwork is satisfactorily completed.
- The vaccine accountability is correct and all used vials have been destroyed.

Audits and Inspections

If necessary, a quality assurance audit will be carried out by independent auditors to make sure that the trial has been conducted according to the protocol and the applicable regulations.

An inspection may be conducted by Regulatory Authorities. The investigator must allow direct access to trial documents.

11 STATISTICAL CONSIDERATIONS

11.1 Determination of the Sample Size

No formal sample size calculation is done for the study and analyses will be mainly descriptive. Given the unknown reactogenicity in this age group and population, it is likely that the immunogenicity of the vaccine will not be statistically significant with this limited study size. Additionally, the numbers of infants will be too small and the variability in infant response to standard immunizations will be too great to make statistically significant conclusions about the interaction between the MVA.HIVA vaccine and the immune response to standard KEPI vaccines.

Statistical Methods

The analyses will be comparative, as clinical and laboratory data from the age-matched vaccine and untreated control groups will be compared.

11.2 Expected analyses and outcomes

11.2.1 Safety of MVA.HIVA

Determined by comparing adverse events, reactogenicity, haematological and biochemical data from the MVA.HIVA recipients and controls. See Tables 2 and 3 for the probabilities of detecting events given the current trial design.

11.2.2 Immunogenicity of MVA.HIVA

Determined by comparing between Groups 1 and 2 and between Groups 3 and 4 the frequency of IFN-γ-producing cells (by ELISPot assay) in response to 15-mer peptides overlapping by 11 amino acids spanning the length of the HIVA immunogen. The MVA.HIVA vaccine will be considered immunogenic in infants if at least 50% of vaccinees have detectable HIV-1-specific T cell responses at any time point after the vaccination.

11.2.3 Immunogenicity of KEPI vaccines

The MVA.HIVA vaccine will be given when the infant is 20 weeks old (at least six weeks apart from another KEPI vaccine) so as to avoid the possibility of vaccine interference. Preliminary data on the possible gross interference of the MVA.HIVA vaccine with the immunogenicity of the KEPI vaccines will be obtained by comparing antibody responses to the KEPI vaccines between Groups 1 and 2 and between Groups 3 and 4.

One of the outcomes of the study is acquisition of preliminary data of any gross interference by MVA.HIVA with the immunogenicity of DTP, HiB, HBV and OPV. Particular focus will be on the immunogenicity of HiB, which is the least immunogenic of these KEPI vaccines and for which antibody levels below 0.5 micrograms per litre are considered unprotective.

11.3 Data Management

A study-dedicated data manager will have overall responsibility for receiving, entering, cleaning, querying, analysing and storing all data that accrues from the study. Most data, including all clinical data, will be entered into the subjects' CRFs and will be transferred by double entry with verification to a secure database. Immunological data will be captured electronically and imported into a separate secure database.

12 SOURCE DOCUMENTS AND ACCESS TO SOURCE DATA/DOCUMENTS

The investigators will maintain appropriate medical and research records for this trial, in compliance with ICH-GCP Guidelines and regulatory and institutional requirements for the protection of confidentiality of subjects. The Principal Investigator, co-investigators and clinical research nurses will have access to records.

The investigators will permit authorized representatives of the Sponsor, Ethics Committees, independent auditors and regulatory agencies to examine clinical records for the purposes of quality assurance reviews, audits and evaluation of the study safety and progress.

All protocol required information will be collected in CRFs designed by the Principal Investigator in collaboration with the data manager. All source documents (e.g., blood result forms, radiology reports, copies of KEPI vaccination cards) will be filed separately and be available for review if needed.

All data on the CRFs must be legibly recorded in blue or black ink or typed. A correction should be made by striking through the incorrect entry with a single line and entering the correct information adjacent to it. The correction must be initialled and dated by the investigator or a designated, qualified individual.

Any requested information that is not obtained as specified in the protocol should have an explanation noted on the CRF as to why the required information was not obtained.

13 QUALITY CONTROL AND QUALITY ASSURANCE

Data will be evaluated for compliance with protocol and accuracy in relation to source documents. The study will be conducted in accordance with procedures identified in the protocol. SOPs will be used at all clinical and laboratory sites. Regular monitoring and an independent audit will be performed according to ICH-GCP Guidelines. Following written standard operating procedures, the monitors will verify that the clinical trial is conducted and data are generated, documented (recorded), and reported in compliance with the protocol, GCP, and the applicable regulatory requirements. The clinical trial site will provide direct access to all trial related sites, source data/documents, and reports for the purpose of monitoring and auditing by the Sponsor, and inspection by local and regulatory authorities.

14 ETHICS/PROTECTION OF HUMAN SUBJECTS

This protocol will be approved by the Kenyatta National Hospital/University of Nairobi Ethics and Research Committee, Oxford Tropical Research Ethics Committee (OXTREC), the University of Washington Institutional Review Board, the Sponsor (MRC), Stockholm Regional Ethics Committee, International AIDS Vaccine Iniative, and the funder (EDCTP) before this study commences. This trial will be conducted in accordance with the current revision of the Declaration of Helsinki and with the International Conference for Harmonisation Good Clinical Practice (ICH-GCP) regulations and guidelines (E6), whichever affords the greater protection to the subject.

14.1 Informed Consent Process

Written informed consent will be obtained from parents of all infants in the study at the screening visit. Fathers will be actively involved in the consent process. The consent form to be used will have been approved by the relevant ethics committees. The trial information sheet, informed consent form, and assessment of understanding questionnaire will be translated into Kiswahili and made available to parents not fluent in English. The subjects may withdraw consent at any time throughout the course of the trial. A copy of the informed consent document will be given to the parents for their records. The rights and welfare of the infants will be protected by emphasizing to the parents that the quality of their medical care will not be adversely affected if they decline to participate in this study.

14.2 Subject Confidentiality

All records will be kept in a locked filing cabinet, which is accessed only by the investigators and the study nurses/field workers. The CRFs will record the infant's 3-digit identification number, and the subject's name or initials will not appear on the forms. All computer entry and networking programs will be done with coded numbers only. Only the investigator's team, the trial monitors, and representatives of the Sponsor, Ethics Committees, independent auditors and regulatory authorities will have access to the records. Photographs taken of vaccination sites (with the subject's written informed consent) will not include the infant's face and will be identified by the infant's 3-digit identification number only. Developed and/or digital photographs will be stored as confidential records as above. This material may be shown to other professional staff, used for educational purposes, or included in a scientific publication. Every effort will be taken to maintain confidentiality.

The study protocol, documentation, data and all other information generated will be held in strict confidence. Clinical information will not be released without written permission of the parents, except as necessary for monitoring.

No information concerning the study or the data will be released to any unauthorized third party, without prior written approval of the Sponsor.

14.3 Biohazard Containment

As the transmission of HIV-1 and other blood-borne pathogens can occur through contact with contaminated needles, blood, and blood products, appropriate blood and secretion precautions will be employed by all personnel involved in the drawing of blood and shipping and handling of all specimens for this study, according to the MRC safety manual.

15 DATA HANDLING AND RECORD KEEPING

The Database Manager, to be named, will be responsible for receiving, entering, cleaning, querying, analysing and storing all data that accrues from the study. S/he will be responsible for linking the epidemiological and clinical data from the field and the clinic with the laboratory data from the immunology, microbiology, haematology and genetics laboratories. The clinical data, and results from haematology, biochemistry, and HIV laboratory tests will be entered into the subject's CRF and transferred to a secure database. Immunological data will, wherever possible, be captured electronically from laboratory machines/computers and imported into a separate database for ultimate linkage with the clinical data. This process will be overseen by the Principal Investigator.

Study Records Retention

Essential documents will be retained until at least 2 years after the last approval of a marketing application in an ICH region and until there are no pending or contemplated marketing applications in an ICH region or at least 2 years have elapsed since the formal discontinuation of clinical development of the investigational product. These documents will be retained for a longer period however if required by the applicable regulatory requirements or by an agreement with the Sponsor. It is the responsibility of the Sponsor to inform the investigator/institution as to when these documents no longer need to be retained

Protocol Deviations

The Principal Investigator (PI) will conduct the trial in compliance with the protocol agreed to by the Sponsor and which was given approval by the relevant Ethics Committees. The Principal Investigator will sign the protocol to confirm agreement.

The PI will not implement any deviation from, or changes to the protocol without agreement by the Sponsor and prior review and documented approval from the relevant Ethics Committees of an amendment, except where necessary to eliminate an immediate hazard(s) to trial subjects, or when the change(s) involve only logistical or administrative aspects of the trial (e.g. change in monitor(s), change of telephone number(s)).

The PI, or person designated by the PI, will document and explain any deviation from the approved protocol on the CRF, where appropriate, and record and explain any deviation in a file note that will be maintained as an essential document. Deviations from the protocol, GCP or trial specific requirements that might have an impact on the conduct of the trial or the safety of participants will be reported within 5 working days to the Sponsor and relevant Ethics Committees, as appropriate.

The investigator may implement a deviation from or a change to a protocol without prior ethics approval *ONLY* to eliminate an immediate hazard(s) to trial subjects. As soon as possible, the implemented deviation or change, the reasons for it, and, if appropriate, the proposed protocol amendment(s) should be submitted to:

A. the Ethics Committee for review and approval and, if required,

B. the Sponsor for agreement, if required.

A protocol deviations folder will contain documentation of all pre-planned deviations from the protocol and their justification.

A protocol violations folder will contain documentation of unplanned protocol violations that occur during the course of the study (e.g., vaccine given at the wrong time).

16 PLANS FOR DISTRIBUTION OF RESEARCH FINDINGS TO STUDY COMMUNITY

16.1 Community Advisory Group

A Community Advisory Group (CAG) will be convened prior to initiating the trial and will involve women and men with HIV-1, including parents of children, community stake-holders and leaders. The study goals, approaches, and methods will be explained at the first meeting in order to determine community issues with the trial. Results will be disseminated to the CAG as they emerge.

At the end of the study, a research report of the methods, detailed results, and brief conclusions will be prepared for distribution by the collaborators. A simplified lay document will be made available to study subjects.

17 REFERENCES

1. Rousseau CM, Nduati RW, Richardson BA, Steele MS, John-Stewart GC, et al. (2003) Longitudinal analysis of human immunodeficiency virus type 1 RNA in breast milk and of its relationship to infant infection and maternal disease J Infect Dis 187: 741-747.
2. Mbori-Ngacha D, Nduati R, John G, Reilly M, Richardson B, et al. (2001) Morbidity and mortality in breastfed and formula-fed infants of HIV-1-infected women: A randomized clinical trial. JAMA 286: 2413-2420.
3. Wherry EJ, Teichgraber V, Becker TC, Masopust D, Kaech SM, et al. (2003) Lineage relationship and protective immunity of memory CD8 T cell subsets Nat Immunol 4: 225-234.
4. Adkins B, Leclerc C, Marshall-Clarke S (2004) Neonatal adaptive immunity comes of age Nat Rev Immunol 4: 553-564.
5. Goulder PJ, Jeena P, Tudor-Williams G, Burchett S (2001) Paediatric HIV infection: correlates of protective immunity and global perspectives in prevention and management Br Med Bull 58: 89-108.
6. Marchant A, Newport M (2000) Prevention of infectious diseases by neonatal and early infantile immunization: prospects for the new millennium Curr Opin Infect Dis 13: 241-246.
7. Siegrist CA (2001) Neonatal and early life vaccinology Vaccine 19: 3331-3346.
8. Buseyne F, Burgard M, Teglas JP, Bui E, Rouzioux C, et al. (1998) Early HIV-specific cytotoxic T lymphocytes and disease progression in children born to HIV-infected mothers AIDS Res Hum Retroviruses 14: 1435-1444.
9. Luzuriaga K, Holmes D, Hereema A, Wong J, Panicali DL, et al. (1995) HIV-1-specific cytotoxic T lymphocyte responses in the first year of life J Immunol 154: 433-443.
10. Pikora CA, Sullivan JL, Panicali D, Luzuriaga K (1997) Early HIV-1 envelope-specific cytotoxic T lymphocyte responses in vertically infected infants J Exp Med 185: 1153-1161.
11. Marchant A, Appay V, Van Der Sande M, Dulphy N, Liesnard C, et al. (2003) Mature CD8(+) T lymphocyte response to viral infection during fetal life J Clin Invest 111: 1747-1755.
12. Hussey GD, Watkins ML, Goddard EA, Gottschalk S, Hughes EJ, et al. (2002) Neonatal mycobacterial specific cytotoxic T-lymphocyte and cytokine profiles in response to distinct BCG vaccination strategies Immunology 105: 314-324.
13. Marchant A, Goetghebuer T, Ota MO, Wolfe I, Ceesay SJ, et al. (1999) Newborns develop a Th1-type immune response to Mycobacterium bovis bacillus Calmette-Guerin vaccination J Immunol 163: 2249-2255.

14. Ota MO, Vekemans J, Schlegel-Haueter SE, Fielding K, Sanneh M, et al. (2002) Influence of Mycobacterium bovis bacillus Calmette-Guerin on antibody and cytokine responses to human neonatal vaccination J Immunol 168: 919-925.
15. Vekemans J, Amedei A, Ota MO, D'Elios MM, Goetghebuer T, et al. (2001) Neonatal bacillus Calmette-Guerin vaccination induces adult-like IFN-gamma production by CD4+ T lymphocytes Eur J Immunol 31: 1531-1535.
16. Marchant A, Goldman M (2005) T cell-mediated immune responses in human newborns: ready to learn? Clin Exp Immunol 141: 10-18.
17. McShane H, Pathan AA, Sander CR, Keating SM, Gilbert SC, et al. (2004) Recombinant modified vaccinia virus Ankara expressing antigen 85A boosts BCG-primed and naturally acquired antimycobacterial immunity in humans Nat Med 10: 1240-1244.
18. Hanke T, McMichael AJ (2000) Design and construction of an experimental HIV-1 vaccine for a year-2000 clinical trial in Kenya. Nat Med 6: 951-955.
19. Slyker JA, Lohman BL, Mbori-Ngacha DA, Reilly M, Wee EG, et al. (2005) Modified vaccinia Ankara expressing HIVA antigen stimulates HIV-1-specific CD8 T cells in ELISpot assays of HIV-1 exposed infants Vaccine 23: 4711-4719.
20. Dorrell L, Yang H, Iversen AK, Conlon C, Suttill A, et al. (2005) Therapeutic immunization of highly active antiretroviral therapy-treated HIV-1-infected patients: safety and immunogenicity of an HIV-1 gag/poly-epitope DNA vaccine Aids 19: 1321-1323.
21. Dorrell L, Yang H, Ondondo B, Dong T, di Gleria K, et al. (2006) Expansion and diversification of HIV-1-specific T cells following immunisation of HIV-1-infected individuals with a recombinant modified vaccinia virus Ankara / HIV-1 gag vaccine. J Virol 80: 4705-4716.
22. Hanke T, Blanchard TJ, Schneider J, Ogg GS, Tan R, et al. (1998) Immunogenicities of intravenous and intramuscular administrations of MVA-based multi-CTL epitope vaccine for HIV in mice. J Gen Virol 79: 83-90.
23. Im EJ, Saubi N, Virgili G, Sander C, Teoh D, et al. (2007) Vaccine platform for prevention of tuberculosis and mother-to-child transmission of human immunodeficiency virus type 1 through breastfeeding J Virol 81: 9408-9418.
24. Letourneau S, Im E-J, Mashishi T, Brereton C, Bridgeman A, et al. (2007) Design and pre-clinical evaluation of a universal HIV-1 vaccine PLoS ONE.
25. Hanke T, Samuel RV, Blanchard TJ, Neumann VC, Allen TM, et al. (1999) Effective induction of simian immunodeficiency virus-specific cytotoxic T lymphocytes in macaques by using a multiepitope gene and DNA prime-modified vaccinia virus Ankara boost vaccination regimen J Virol 73: 7524-7532.
26. Im E-J, di Gleria K, McMichael AJ, Hanke T (2006) Induction of long-lasting multi-specific $CD8^+$ T cells by a 4-component DNA-MVA/HIVA-RENTA candidate HIV-1 vaccine in rhesus macaques Eur J Immunol 36: 2574-2584.
27. Robinson HL, Montefiori DC, Johnson RP, Manson KH, Kalish ML, et al. (1999) Neutralizing antibody-independent containment of immunodeficiency virus challenges by DNA priming and recombinant pox virus booster immunizations Nat Med 5: 526-534.
28. Seth A, Ourmanov I, Schmitz JE, Kuroda MJ, Lifton MA, et al. (2000) Immunization with a modified vaccinia virus expressing simian immunodeficiency virus (SIV) Gag-Pol primes for an anamnestic Gag-specific cytotoxic T-lymphocyte response and is associated with reduction of viremia after SIV challenge. J Virol 74: 2502-2509.
29. Amara RR, Villinger F, Altman JD, Lydy SL, O'Neil SP, et al. (2001) Control of a Mucosal Challenge and Prevention of AIDS by a Multiprotein DNA/MVA Vaccine Science 292: 69-74.
30. Stittelaar KJ, Kuiken T, de Swart RL, van Amerongen G, Vos HW, et al. (2001) Safety of modified vaccinia virus Ankara (MVA) in immune-suppressed macaques Vaccine 19: 3700-3709.
31. Hanke T, McMichael AJ, Samuel RS, Powell LAJ, McLoughlin L, et al. (2002) Lack

of toxicity and persistence in the mouse associated with administration of candidate DNA- and modified vaccinia virus Ankara (MVA)-based HIV vaccines for Kenya Vaccine 21: 109-115.
32. Hanke T, McMichael AJ, Dennise MJ, Sharpe SA, Powell LAJ, et al. (2005) Biodistribution and persistence of an MVA-vectored candidate HIV vaccine in SIV-infected rhesus macaques and SCID mice. Vaccine 23: 1507-1514.
33. Estcourt MJ, Letourneau S, McMichael AJ, Hanke T (2005) Vaccine route, dose and type of delivery vector determine patterns of primary CD8+ T cell responses Eur J Immunol 35: 2532-2540.
34. Estcourt MJ, McMichael AJ, Hanke T (2005) Altered primary CD8(+) T cell response to a modified virus Ankara(MVA)-vectored vaccine in the absence of CD4(+) T cell help Eur J Immunol.
35. Hanke T, Barnfield C, Wee EG-T, Ågren L, Samuel RV, et al. (2003) Construction and immunogenicity in a prime-boost regimen of a Semliki Forest virus-vectored experimental HIV clade A vaccine J Gen Virol 84: 361-368.
36. Larke N, Murphy A, Wirblich C, Teoh D, Estcourt MJ, et al. (2005) Induction of human immunodeficiency virus type 1-specific T cells by a bluetongue virus tubule-vectored vaccine prime-recombinant modified virus Ankara boost regimen J Virol 79: 14822-14833.
37. Nkolola JP, Wee EG-T, Im E-J, Jewell CP, Chen N, et al. (2004) Engineering RENTA, a DNA prime-MVA boost HIV vaccine tailored for Eastern and Central Africa Gene Ther 11: 1068-1080.
38. Nordstrom EK, Forsell MN, Barnfield C, Bonin E, Hanke T, et al. (2005) Enhanced immunogenicity using an alphavirus replicon DNA vaccine against human immunodeficiency virus type 1 J Gen Virol 86: 349-354.
39. Wee EG-T, Patel S, McMichael AJ, Hanke T (2002) A DNA/MVA-based candidate HIV vaccine for Kenya induces multi-specific T cell responses in rhesus macaques. J Gen Virol 83: 75-80.
40. Mayr A, Hochstein-Mintzel V, Stickl H (1975) Abstammung, Eigenschaften und Verwendung des attenuierten Vaccinia-Stammes MVA. Infection 105: 6-14.
41. Mayr A, Stickl H, Muller HK, Danner K, Singer H (1978) Der Pockenimpfstamm MVA: Marker, genetische Struktur, Erfahrungen mit der parenteralen Schutzimpfung und Verhalten im abwehrgeschwachten Organismus. Zentralbl Bakt Hyg I (Abt Orig B) 167: 375-390.
42. Bejon P, Peshu N, Gilbert SC, Lowe BS, Molyneux CS, et al. (2006) Safety profile of the viral vectors of attenuated fowlpox strain FP9 and modified vaccinia virus Ankara recombinant for either of 2 preerythrocytic malaria antigens, ME-TRAP or the circumsporozoite protein, in children and adults in Kenya Clin Infect Dis 42: 1102-1110.
43. Cosma A, Nagaraj R, Buhler S, Hinkula J, Busch DH, et al. (2003) Therapeutic vaccination with MVA-HIV-1 nef elicits Nef-specific T-helper cell responses in chronically HIV-1 infected individuals Vaccine 22: 21-29.
44. Harrer E, Bauerle M, Ferstl B, Chaplin P, Petzold B, et al. (2005) Therapeutic vaccination of HIV-1-infected patients on HAART with a recombinant HIV-1 nef-expressing MVA: safety, immunogenicity and influence on viral load during treatment interruption Antivir Ther 10: 285-300.
45. Gilbert SC, Moorthy VS, Andrews L, Pathan AA, McConkey SJ, et al. (2006) Synergistic DNA-MVA prime-boost vaccination regimes for malaria and tuberculosis Vaccine 24: 4554-4561.
46. Im E-J, Hanke T (2004) MVA as a vector for vaccines against HIV-1 Expert Rev Vaccines 3: S89-97.
47. Sutter G, Staib C (2003) Vaccinia vectors as candidate vaccines: the development of modified vaccinia virus Ankara for antigen delivery Curr Drug Targets Infect Disord 3: 263-271.

48. Bejon P, Mwacharo J, Kai O, Mwangi T, Milligan P, et al. (2006) A phase 2b randomised trial of the candidate malaria vaccines FP9 ME-TRAP and MVA ME-TRAP among children in Kenya PLoS Clin Trials 1: e29.
49. Bejon P, Mwacharo J, Kai OK, Todryk S, Keating S, et al. (2006) Immunogenicity of the candidate malaria vaccines FP9 and modified vaccinia virus Ankara encoding the pre-erythrocytic antigen ME-TRAP in 1-6 year old children in a malaria endemic area Vaccine 24: 4709-4715.
50. Greenough TC, Cunningham CK, Muresan P, McManus M, Persaud D, et al. (2008) Safety and immunogenicity of recombinant poxvirus HIV-1 vaccines in young adults on highly active antiretroviral therapy Vaccine 26: 6883-6893.
51. Bart PA, Goodall R, Barber T, Harari A, Guimaraes-Walker A, et al. (2008) EV01: a phase I trial in healthy HIV negative volunteers to evaluate a clade C HIV vaccine, NYVAC-C undertaken by the EuroVacc Consortium Vaccine 26: 3153-3161.
52. de Bruyn G, Rossini AJ, Chiu YL, Holman D, Elizaga ML, et al. (2004) Safety profile of recombinant canarypox HIV vaccines Vaccine 22: 704-713.
53. Kanesa-thasan N, Smucny JJ, Hoke CH, Marks DH, Konishi E, et al. (2000) Safety and immunogenicity of NYVAC-JEV and ALVAC-JEV attenuated recombinant Japanese encephalitis virus--poxvirus vaccines in vaccinia-nonimmune and vaccinia-immune humans Vaccine 19: 483-491.
54. McCormack S, Stohr W, Barber T, Bart PA, Harari A, et al. (2008) EV02: a Phase I trial to compare the safety and immunogenicity of HIV DNA-C prime-NYVAC-C boost to NYVAC-C alone Vaccine 26: 3162-3174.
55. Ockenhouse CF, Sun PF, Lanar DE, Wellde BT, Hall BT, et al. (1998) Phase I/IIa safety, immunogenicity, and efficacy trial of NYVAC-Pf7, a pox-vectored, multiantigen, multistage vaccine candidate for Plasmodium falciparum malaria J Infect Dis 177: 1664-1673.
56. McFarland EJ, Johnson DC, Muresan P, Fenton T, Tomaras GD, et al. (2006) HIV-1 vaccine induced immune responses in newborns of HIV-1 infected mothers Aids 20: 1481-1489.
57. Hanke T, McMichael AJ, Dorrell L (2007) Clinical experience with plasmid DNA- and modified vaccinia vaccine Ankara (MVA)-vectored HIV-1 clade A vaccine inducing T cells J Gen Virol 88: 1-12.
58. Cebere I, Dorrell L, McShane H, Simmons A, McCormack S, et al. (2006) Phase I clinical trial safety of DNA- and modified virus Ankara-vectored human immunodeficiency virus type 1 (HIV-1) vaccines administered alone and in a prime-boost regime to healthy HIV-1-uninfected volunteers Vaccine 24: 417-425.
59. Yang H, Dong T, Turnbull E, Ranasinghe S, Ondondo B, et al. (2007) Broad TCR usage in functional HIV-1-specific CD8+ T cell expansions driven by vaccination during highly active antiretroviral therapy J Immunol 179: 597-606.
60. Goonetilleke N, Moore S, Dally L, Winstone N, Mahmoud N, et al. (2006) Prime-boost vaccination with recombinant DNA and MVA expressing HIV-1 Clade A gag and immunodominant CTL epitopes induces multi-functional HIV-1-specific T cells in healthy subjects J Virol 80: 4717-4728.

18 APPENDIX 1 – Clinical Trial Information Sheet

Medical Research Council MRC

Study PV002

AN OPEN RANDOMIZED PHASE I/II STUDY EVALUATING SAFETY AND IMMUNOGENICITY OF A CANDIDATE HIV-1 VACCINE, MVA.HIVA, ADMINISTERED TO HEALTHY INFANTS BORN TO HIV-1-INFECTED MOTHERS

What is this vaccine trial for?

AIDS is a devastating disease caused by the HIV virus. This trial will test a vaccine being developed against AIDS, called MVA.HIVA, which is intended to prevent infection of infants born to HIV-infected mothers by HIV in mother's breast milk. Right now, mothers who are HIV-infected can take medicines called antiretrovirals to reduce the chance that their infant will become infected with HIV during delivery or breastfeeding. Even with these drugs, there is still a risk of infecting infants through breast milk. Many HIV-infected women still chose to breastfeed their infants because mother's milk has many other healthy benefits that formula milk doesn't have. We hope that a vaccine against AIDS would help lower the risk of HIV-infection through breast milk.

We have already tested this vaccine in around 400 healthy, non-HIV-infected and HIV-infected adults in the UK, Europe and Africa. Now we would like to test the vaccine in infants in Kenya. We need to do this because if it were shown to work well, the vaccine would eventually be used in many children born to HIV-infected mothers to prevent the spread of HIV. This study wants to make sure that this new vaccine does not interfere with any other children vaccines, that it has the effect we expect on the immune system, and that there are no unwanted effects caused by this vaccine.

How will I and my child be recruited?

You, as a mother expecting a new child and attending an antenatal clinic that is a recruitment site for this study, are being approached about enrolling in this vaccine study. You may want to/should consult with the child's father before you enrol. If you are willing to participate, we ask that you come to Kenyatta National Hospital during your second trimester or early in your third trimester so we can explain the study one more time to you and ask you again if you wish to be a part of this study. If you do, you will be asked to read and sign (or thumb-print) the informed consent form.

What does taking part involve for me?

You will be asked to go through a screening process as part of the enrolment in the study. This screening includes taking up to 5 ml (a maximum of one teaspoon) of your blood for an HIV test and to count your CD4 cells, a physical examination to make sure you are healthy enough to participate, and answering some general questions about your job and living situation. You will also be asked to have a second CD4 test 6 weeks after your delivery.

After your screening, you will be referred to the antenatal care clinic at Kenyatta Nationals Hospital, where you will have to register as a client to receive medication to prevent HIV transmission to your child during pregnancy and delivery. This type of medicine is called highly active antiretroviral therapy, or HAART. If you breastfeed your child, you will receive this medication during breastfeeding from the Comprehensive Care Clinic at Kenyatta National Hospital.

You will be asked to deliver your baby at Kenyatta National Hospital. Your delivery and any related medical fees will be paid for by the study. After your infant is born, if he/she meets all the eligibility requirements and is enrolled, you will be asked to bring your child to the study clinic at Kenyatta National Hospital for each of the 10 study visits.

You will also be asked to decide whether you wish to breastfeed or formula-feed your child. The study will provide information about the risks and benefits of either choice. Formula will be provided to you for free if you choose to formula-feed your child.

What does taking part involve for my child?
We want to make sure your child is well before giving him/her the vaccine. To check this we need to carry out some examinations, including a number of blood tests. This will include tests for anaemia, and kidney or liver problems. If needed, we will tell you about treatment for any problems we discover.

Your child will join either a vaccine or control group of the study. If your child is in the vaccine group, he/she will receive one injection of the study vaccine, called MVA.HIVA, in addition to the routine KEPI childhood vaccines. Children put into the control group will not receive any extra injection in addition to the standard KEPI regimen.

We will ask you to bring your child to the clinic at 6, 10, 14, and 36 weeks of age for his/her regular KEPI vaccinations. We will also ask you to bring your child to the clinic at 2, 19, 20, 21, 28, and 48 weeks of age for study follow-up visits. The doctor will check for any unwanted reactions like redness or itching at the injection site on all clinic visits after vaccination. A staff member will visit every child at home for the first two days after the injection of the study. The staff member will not wear a uniform and will take care not to let other people know you and your child are in this study.

At every clinic visit, your child will be tested for HIV. This will usually involve pricking the heel of your infant's foot to collect blood drops for the test. Children who are helping to test this vaccine will also have blood tests using 3 – 5 ml (a maximum one teaspoon) of blood collected on 5 occasions, at least 2 weeks apart. This volume of blood is safe, and children are able to replace the blood themselves if they are healthy.

Blood tests are necessary to test the safety of the vaccine, and to learn about how your child's blood changes and responds to the vaccines. We will want to store some of the blood to do some later tests related to how this study vaccine works. We will also do a genetic test to better understand how your child's immune system can respond to the vaccine. We may ask to photograph the injection site to record any side effects.

Schedule of vaccinations and bleeds at study visits

Child's Age	3rd Tri-mester	Birth	6 w	10 w	14 w	19 w	20 w	21 w	28 w	36 w	48 w
Study visit	Screen-ing	Enrol-ment	2	3	4	5	6	7	8	9	10
Vaccine Group	**M.Bleed**	**I.Prick** KEPI	**I.Prick** **M.Bleed** KEPI	**I.Prick** KEPI	**I.Prick** KEPI	**I.Bleed**	**MVA.HIVA** **I.Prick**	**I.Bleed**	**I.Bleed**	**I.Bleed** KEPI	**I.Bleed**
Control Group	**M.Bleed**	**I.Prick** KEPI	**I.Prick** **M.Bleed** KEPI	**I.Prick** KEPI	**I.Prick** KEPI	**I.Bleed**	**I.Prick**	**I.Bleed**	**I.Bleed**	**I.Bleed** KEPI	**I.Bleed**

M.Bleed = up to one teaspoon of mother blood collected; I.Bleed = up to one teaspoon of infant blood collected; I.Prick = drops of blood from infant's heel collected; MVA.HIVA = study vaccine administered to infant; KEPI = childhood vaccines of the Kenyan Expanded Programme on Immunization given; w = week

Is there any risk to my child if they take part in this vaccine trial?

Children will need to stay at the clinic for one hour after the study vaccine injection so that they can be watched for unwanted or unexpected reactions. Although very rare, serious reactions can happen with any vaccination. These might include skin swelling, shortness of breath and light-headedness or fainting. Medical equipment necessary to treat any serious reactions will be available. We estimate that severe unwanted reactions will happen for 1 in a million vaccinations. Although we do not think these serious reactions will happen to your child, we are prepared to treat them if they do. By an insurance policy, we will be responsible to provide for treatment and damages costs caused by the vaccine.

Mild reactions were common when this vaccine was tested in other studies. As with any injection, children will feel pain at the injection site, but this will not last long. The most common side effect is the skin changing colour around the site of injection a few days after the vaccination. Occasionally, there is some scaling, which we expect will go away within 2 weeks.

The vaccine cannot cause HIV infection or AIDS. We don't know yet how well the vaccine will work to prevent HIV transmission, so there is a risk of HIV infection through breast milk and delivery. The study will provide antiretroviral medicine to you to help prevent HIV infection during pregnancy and delivery and if you choose to breastfeed. We will test your child for HIV at every study visit. There is a very small chance that the result may come back as positive due to the receipt of the study vaccine, even if the child is not infected with HIV. If an HIV test result comes back as positive, we will run a different type of test to see if the result is because the child is infected or because the study vaccine has changed the test result. If the test is positive at the end of the study, but the child is not infected with HIV, we will provide you with a card to explain that your child has taken part in an HIV vaccine study and that some routine HIV tests might be positive even though the child is not infected. Please always contact the study team first before your child undergoes an HIV test outside the study centre.

What if I decide not to involve my child or change my mind later?
You do not have to agree for your child to take part in this trial and even if you agree you can change your mind and withdraw from the trial at any time. If you decide you do not wish your child to take part now, or change your mind later, the regular medical treatment available to your child will not change or be affected in any way.

What if I have any more questions?
You are free to ask any questions you wish now, or at any stage during the trial to the field worker or doctor. You should be happy that all your questions have been answered fully.

The Kenyatta National Hospital/University of Nairobi Ethics and Research Committee has approved this study. All information collected for this study will be treated confidentially.

If you are willing to help us with this study, please return to the clinic in one week's time and before your delivery so that we can enroll you and your infant into this study. At that visit, we would be happy to answer any more questions you may have. If you require further information please contact Associate Professor Walter Jaoko (contact information below).

Thank you very much.
Professor Walter Jaoko, MB, ChB, MTropMed, PhD, Site Principal Investigator
Department of Medical Microbiology, University of Nairobi
Second Floor, PO Box 19676, Off Ngong Road, Nairobi, Kenya
Phone: 254-02-2717694 / 275404 Email: Wjaoko@kaviuon.org

19 APPENDIX 2 – Consent Form Version 1.9

University of Washington
Oxford Tropical Research Ethics Committee
Kenyatta National Hospital/University of Nairobi

INFORMED CONSENT FORM

Version 1.9

An Open Randomized Phase I/II Study Evaluating Safety and Immunogenicity of a Candidate HIV-1 Vaccine, MVA.HIVA, Administered to Healthy Infants Born to HIV-1 Infected Mothers (PedVac002)
Sponsor: Medical Research Council (MRC) Project code: GC_ct_06_33111

Researchers:

- Walter Jaoko, MB, ChB, MTropMed, PhD; **Site Principal Investigator**, Department of Medical Microbiology, University of Nairobi +254-02-2717694
- Tomas Hanke, BSc, MSc, PhD; **Chief Investigator**, Weatherall Institute of Molecular Medicine, University of Oxford +44 (0) 1865-222355
- Grace John-Stewart, MD, PhD; **Co-Investigator**, Department of Medicine, University of Washington 206-543-4278
- Sarah Rowland-Jones, MBChB, MMed, PhD; **Co-Investigator**; MRC Laboratories, The Gambia +220-449-6188
- Dalton Wamalwa, MMed, MPH; **Co-Investigator**; Department of Paediatrics and Child Care, Kenyatta National Hospital, University of Nairobi +254-02-2731498
- Ruth Nduati, MMed, MPH; **Co-Investigator**; Department of Paediatrics, University of Nairobi +254-20-2714851
- Dorothy Mbori-Ngacha, MMed, MPH; **Co-Investigator**; Department of Paediatrics, University of Nairobi +254-20-2720947
- Elizabeth Obimbo, MMed, MPH; **Co-Investigator**; Department of Paediatrics, University of Nairobi +254-20-2731974
- Omu Anzala, PhD, MBChB; **Co-Investigator**; Department of Medical Microbiology, University of Nairobi +254-20-2717694
- Barbra Richardson, PhD; **Co-Investigator**; Department of Biostatistics, University of Washington 206-731-2425
- Carey Farquhar, MD, PhD; **Co-Investigator**; Department of Medicine, University of Washington 206-543-4278
- Barbara Lohman-Payne, PhD; **Co-Investigator**; Department of Paediatrics, University of Washington +254-20-2726300
- Marie Reilly, MSc, PhD; **Co-Investigator**; Department of Medical Epidemiology and Biostatistics, Karolinska Institutet +46-8-524-80000
- Gwen Ambler, MPH; **Co-Investigator**, Department of Medicine, University of Washington 206-543-4278

24-Hour Emergency Telephone Number: Kenyatta National Hospital, +254-20-2726300
Dalton Wamalwa, MMed, MPH, +254-721239493 (cellphone)

RESEARCHER'S STATEMENT

What is a consent form?

We are asking you and your expected child to be in a research study. The purpose of this consent form is to give you the information you will need to help you decide whether you agree that you and your child will be in the study or not. Please read this form carefully. You may ask questions about the purpose of the research, what we will ask you to do, the possible risks and benefits, your rights as a volunteer, and anything else about the research or this form that is not clear. When we have

answered all of your questions, you can decide if you want to be in the study or not. This process is called "informed consent." We will give you a copy of this form for your records.

This consent form may have words that you do not understand. Please ask the researcher or the study staff to explain any words or information that you do not clearly understand. You may take home an unsigned copy of this consent form to think about or discuss with family or friends before making your decision.

What is the purpose of the study?

Many infants born to HIV-positive women become infected with the HIV virus either during pregnancy or labour, or through their mother's breast-milk. The purpose of this trial is to see 1) how well an AIDS vaccine candidate (known as MVA.HIVA) is tolerated in infants, 2) whether it has the expected effect on infant immune systems, and 3) whether it interferes with any other childhood vaccines (like pertussis, diphtheria, hepatitis B, and measles vaccines). This study is for women who are HIV-positive and their uninfected newborn infants.

What is the study vaccine?

The study vaccine is called MVA.HIVA. Almost 750 doses of this vaccine have already been tested in approximately 400 healthy, non-HIV-infected and HIV-infected adults in the UK, Europe and Africa. In all these tests, there were no reports of serious health problems. In these healthy subjects, the studies show that the MVA.HIVA vaccine is generally safe and well tolerated in the short-term and medium-term. It is absolutely NOT POSSIBLE to get HIV infection from the vaccine.

We would now like to test the vaccine in 36 infants in Kenya. We need to do this so that we can better understand how the vaccine works with infants' immune systems. This information will be important for improving and developing the vaccine to be effective at preventing HIV infection in the future.

Procedures

If you agree that you and your child will participate in this study and you both meet all of the study eligibility requirements, we will ask you to agree to the following things:

Mother
- A screening examination
- Start an antiretroviral therapy regimen during pregnancy
- An enrolment questionnaire
- Delivery at Kenyatta National Hospital (KNH)
- Accept a home visit by a community worker for two days after the study vaccine injection

Child
- Return to KNH for study visits when at 2, 6, 10, 14, 19, 20, 21, 28, 36, and 48 weeks of age
- Collection of one teaspoon of blood during 5 different study visits
- Receiving all KEPI childhood vaccines at birth and during 4 of the study visits
- Randomization to study vaccine group or control group at 20 weeks of age
- Vaccination for those in study vaccine group at 20 weeks of age

Each of these procedures is described below.

Mother

a. Screening examination: As part of being enrolled in the study, we will ask you to answer some questions about your medical history, have up to one teaspoon of your blood drawn for an HIV test and CD4 count, and have a physical examination to make sure you are healthy enough to participate. You will also be asked to have a second CD4 count 6 weeks after your delivery. These procedures are part of the screening process and are necessary to make sure that you qualify for the study.
b. Start an antiretroviral therapy regimen during pregnancy: You will be referred to clinics at Kenyatta National Hospital to receive medication that will help prevent mother-to-child transmission of HIV. This treatment is called highly active antiretroviral therapy, or HAART, and

involves taking daily pills during your pregnancy, delivery, and period of breastfeeding (if you are breastfeeding).

c. Enrolment questionnaire: If the screening results show that you are eligible to continue with the study, we will ask you to complete a survey before you give birth. We will ask you some questions about your living situation, including whether you are married, what sources of income your family has, and whether you plan on breast-feeding or formula-feeding your child. You may refuse to answer any questions.
d. Delivery at Kenyatta National Hospital (KNH): We will ask you to return to KNH to deliver your child. You will receive standard care during your labour and delivery. The expense of your delivery will be paid for by the study. At delivery, your child will receive a physical examination to make sure that he/she is well before being enrolled in this study.
e. Accept home visits by a community worker: A member of staff will visit every child at home for the first two days after the study vaccine injection. During these two visits after the injection, the staff nurse will check for any unwanted reactions, like redness, swelling or tenderness at the injection site. The staff member will not wear a uniform and will take care not to let other people know you and your child are in this study.

Child

f. Return to KNH for 10 study visits: We will ask you to return to the KNH clinic for study visits when your child is 2, 6, 10, 14, 19, 20, 21, 28, 36, and 48 weeks old. You will be compensated Ksh 300 for travel expenses each time you come to the clinic for these 10 study visits. At each study visit, you will be asked questions about the health of your child, he/she will be examined by a doctor, and he/she will have blood collected. At some of these study visits, you child will also receive his/her KEPI childhood vaccinations. If your child is randomised to receive the study vaccination, it will be injected into the child's arm at the 20 week visit. For any study visit following vaccination with the study vaccine, a doctor will check for any other unwanted reactions like redness, swelling or tenderness at the injection site. We may also ask to photograph the injection site to record any side effects.
g. Collection of blood: At 5 of the 10 study visits, children will have blood tests using 3 – 5 ml (maximum one teaspoon) of blood collected by a trained nurse using sterile techniques. These bleedings are at least 2 weeks apart. This volume of blood is safe, and children are able to replace the blood themselves if they are healthy. We will do blood tests to test the safety of the vaccine and to learn about how your child's blood changes and responds to the vaccines. We will want to store some of the blood to do some tests later to see how this study vaccine works. We shall seek approval from the Ethical Committee before using samples for further studies. Blood test will also include a test for anaemia and kidney or liver problems. If needed, we will tell you about treatment for any problems we discover. Your child will also have his/her heel pricked to collect drops of blood for an HIV test. This blood will be collected at birth and at 4 of the other study visits.
h. Receive all KEPI childhood vaccinations: At birth and the study visits when your child is 6, 10, 14, and 36 weeks of age, your child will receive his/her regular KEPI childhood vaccinations (see table at left). There will be a sticker placed in your child's immunization booklet indicating that your child is part of a study and that he/she should only receive KEPI vaccinations from study staff.

Age	
Birth	Tuberculosis (BCG), polio (OPV)
6 weeks	Diphtheria, tetanus, pertussis (DTP), meningitis (HiB), hepatitis B (HBV), OPV
10 weeks	DTP, HiB, HBV, OPV
14 weeks	DTP, HiB, HBV, OPV
9 months	Measles

i. Randomisation to study vaccine group or control group: At age 20 weeks, your child will be put in either a vaccine or control group of the study. If your child is in the vaccine group, he/she will receive one single injection of the study vaccine, called MVA.HIVA. If your child is in the control group, he/she will not receive the study vaccine.
j. Vaccination for those in study vaccine group at 20 weeks of age: Before receiving the study vaccine, your child will be examined to make sure that he/she is healthy and meets all of the eligibility criteria to receive the vaccine. If your child meets all the requirements to continue with the study, the study vaccine will be injected into the child's left arm muscle and the child will be watched for 1 hour after the injection to make sure he/she does not have any reactions.

It is very important that you and your child come for all your scheduled study visits. If you miss a visit, the study staff will try to contact you. They will do this by first calling you and then by visiting your home and trying to find you. They may also talk to the contact people that you list. If they talk to these people, they will not tell them why they are trying to reach you.

Schedule of vaccinations and bleeds at study visits:

Child's Age	3rd Tri-mester	Birth	6 w	10 w	14 w	19 w	20 w	21 w	28 w	36 w	48 w
Study visit	Screen-ing	Enrol-ment	2	3	4	5	6	7	8	9	10
Vaccine Group	**M.Bleed**	**I.Prick** KEPI	**I.Prick M.Bleed** KEPI	**I.Prick** KEPI	**I.Prick** KEPI	**I.Bleed**	**MVA.HIVA I.Prick**	**I.Bleed**	**I.Bleed**	**I.Bleed** KEPI	**I.Bleed**
Control Group	**M.Bleed**	**I.Prick** KEPI	**I.Prick M.Bleed** KEPI	**I.Prick** KEPI	**I.Prick** KEPI	**I.Bleed**	**I.Prick**	**I.Bleed**	**I.Bleed**	**I.Bleed** KEPI	**I.Bleed**

M.Bleed = up to one teaspoon of mother blood collected; **I.Bleed** = up to one teaspoon of infant blood collected; **I.Prick** = infant's finger pricked; MVA.HIVA = study vaccine administered to infant; KEPI = childhood vaccines of the Kenyan Expanded Programme on Immunization given; w = week

Risks and Discomfort

We do not anticipate that will be any serious adverse effects due to the study vaccine. However, as with any vaccine, there may be side effects, including the risk of death, that we do not know about. There may also be unanticipated or unexpected risks, including long-term risks that may not be known until later, and sometimes not for many years after the study is over. Children will need to stay at the clinic for one hour after the study vaccine injection so that they can be observed for unwanted or unexpected reactions. Although very rare, serious reactions can occur with any vaccination. These include skin swelling, shortness of breath and light-headedness or fainting. Medical equipment necessary to treat any serious reactions will be available. We estimate that severe unwanted reactions will happen for 1 in a million vaccinations. Although we do not think these serious reactions will happen, we are prepared to treat them, and by an insurance policy, we will be responsible to provide for treatment and damages costs determined to be probably caused by the vaccine.

Mild reactions were common when these vaccines were tested in other studies. As with any vaccination, children will feel pain at the injection site, but this will not last long. The most common side effect is the skin changing colour at the site of injection a few days after the vaccination. Occasionally, there is some scaling and itching, which we expect will go away within 2 weeks.

Having blood drawn may cause some pain to your child. A bruise may form where the needle enters the vein. Drawing blood may cause some people to faint.

The vaccine cannot cause HIV infection or AIDS. We don't know yet whether or not the vaccine will work to prevent HIV transmission through breast milk, so there is a risk that your baby will get infected with HIV if you breastfeed, and if so, will not be enrolled. Your baby may have already become infected with HIV before birth or during delivery. This study is not designed to test whether this vaccine can prevent HIV infection at birth. The study will provide antiretroviral medication to you to help prevent HIV infection during pregnancy and delivery and while breastfeeding, if you choose to do so. We will test your child for HIV at every study visit. Some people experience side effects from taking antiretroviral medication. Your doctor will discuss the potential effects of the medication you are prescribed and will monitor your health closely while you are taking the antiretrovirals.

If your child is HIV negative at birth, he/she may still test positive for HIV at delivery because he/she will have your antibodies to HIV. After receiving the study vaccination at 20 weeks, there is a very small chance that an HIV test result may come back as positive due to the receipt of the study vaccine, even if the child is not infected with HIV. If an HIV test result comes back as positive, we will run a different type of test to see if the result is because the child is infected or because the study vaccine has influenced the test. If the test is positive at the end of the study, but the child is not

infected with HIV, we will provide you with a card to explain that your child has taken part in an HIV vaccine study and that some routine HIV tests might be positive even though the child is not infected. Please always contact the study team first before your child undergoes an HIV test outside the study centre.

Your child will be randomly assigned to receive the study vaccine or no additional vaccine aside from the KEPI childhood vaccinations. The vaccines your child will receive may prove to be less effective or have more side effects than the other study treatment.

Alternatives to Taking Part in this Study

There are alternatives to being in this study. You can follow national guidelines for feeding and caring for your child to help avoid his/her becoming infected with HIV. These guidelines recommend exclusive formula feeding in situations where you do not want to breast-feed, can afford to consistently buy formula, and have access to clean drinking water to mix it with. If those requirements are not met, the national guidelines recommend exclusively breast-feeding until your child is three to six months old. Through the antenatal clinic, post pregnancy care clinic, and Comprehensive Care Clinic at KNH, you can access antiretroviral drugs to prevent HIV infection in your child even if you do not participate in this study. Being in this study is voluntary. You may choose not to be in this study, and you may withdraw from the study at any time without penalty or loss of benefits to which you are otherwise entitled.

Benefits of the Study

This study offers the possibility that your child could benefit from the vaccine. However, it is very unlikely that there will be any benefit. We hope that the results of this study will help in the vaccination of future children against HIV infection.

Other Information

Costs to You
There is no cost to you for being in this study. Vaccines available to you from the study will be given free of charge.

Reimbursement
You will receive payment for the costs of travel to your clinic visits. At each scheduled clinic visit you will receive KSh 300 to reimburse you for travel expenses. If you come to clinic for a visit because of problems, illness or side-effects you will receive a travel reimbursement of KSh 300. As part of the study, you will receive free delivery and antenatal care at Kenyatta National Hospital.

Confidentiality
Efforts will be made to keep all personal information related to you and your child confidential. We will create a random study identification number for each person in the study and will label all study data with this number and not your name. The files of the individuals in this study will be stored in a room that is accessible only to the study researchers and is under lock and key. The link between your infant's identifying information (like name and address) and study identification number will be destroyed around 2 years after the study is completed. Any publication of this study will not use your name or your infant's name or identify you or your infant personally.

In addition to the researchers, the following groups may have access to study data which may include identifiable health information from your medical records:

- University of Washington (UW) Human Subjects Review Committee (the board that oversees research at the UW);
- UW Medicine's compliance and quality improvement groups;
- Medical Research Council, the sponsor of this study;
- Oxford Tropical Research Ethics Committee (OXTREC) (the board that oversees research at Oxford University);

- Kenyatta National Hospital/University of Nairobi ethics and research committee (the board that oversees research at KNH);
- Data and safety monitoring boards that are responsible for the safety of research subjects;

Any reviewer from one of these organizations will protect your privacy if your records are examined. Although we will make every effort to keep your information confidential, no system for protecting your confidentiality can be completely secure. It is possible that unauthorized persons might discover that you are in this study, or might obtain information about you.

We will put a copy of this consent form in your medical record. We will also put copies of test and exam results from this study into your medical record if the results are important for your medical care. The results of your screening tests may show that you are not eligible to continue in the study. If this happens, we will keep your screening information until February 28, 2011 and then we will destroy the information.

Research-Related Injury
If you think your child has an injury or illness related to this study, contact the study staff right away. If your child is injured as a result of being in the study, the study staff will give you immediate necessary treatment for the injuries, free of charge. The Medical Research Council will pay to treat injury or illness determined to be definitely or probably caused by the study vaccine. The study staff also will tell you where you can get additional treatment for the injuries, if needed. You will not lose your legal right to seek payment if you sign this form.

Withdrawal
At any time you are free to withdraw or refuse to participate in the research study. Additionally, we may withdraw you from this study without your consent if we believe it is in your best interest, if you are unable to follow study procedures, or if you or your child no longer meet all the study eligibility requirements.

If we learn new information about the risks or benefits of participating in this study that would potentially affect your decision to stay in the study, we will notify you.

Problems or Questions
If you ever have any questions about the study you should contact Dr. Dalton Wamalwa at KNH extension 43650 or 2733087 or 0710344159. If you have questions about your rights as a research participant, you should contact Professor Bhatt, the Chair of the Kenyatta National Hospital/University of Nairobi Ethics and Research Committee, at 2726300-9 ext. 44102.

Specimen Storage and Use of your Samples for Future Studies
We would like to save samples of your infant's blood at the University of Nairobi and University of Washington for future research by us and by other researchers. We will use these samples for research related to how infants' immune systems react to KEPI childhood vaccines and to vaccines aimed at preventing HIV infection as well as how infants fight infections ("immunity"). This will include testing for genes which may affect whether a person is more or less likely to get these infections. Other research questions that could be answered using the stored samples include: looking at your infant's and your own immune responses to infections that are common in Kenya, such as tuberculosis and cytomegalovirus; your infant's immune responses to vaccinations that children in Kenya are routinely given; genetic characteristics of you and your infant and whether these characteristics make you or your infant more or less likely to get certain infections; and how the immune responses and genetic characteristics in your infant and you are related. This research is experimental and these tests are not useful for you or your infant's clinical care. Before your infant's samples leave the clinic, they will be assigned a code and your infant's name will not be on them. Your infant's name will be linked to the code only for five years after the study is completed. After that time, the link between your infant's name and the code on the samples will be destroyed. An Institutional Review Board or Independent Ethics Committee, which watches over the safety and rights of research participants, must approve any future research studies using your or your infant's samples. If you agree to store your infant's samples, we will keep them in unidentifiable form (not linked to your infant's name) indefinitely or for as long as there is sample that can be used for future research. If you do not want to have your infant's samples stored for future research, you can still be

in this study and your infant's samples will be destroyed once testing for the study is completed. If you agree to the storage of your infant's samples now, but change your mind before the end of the study, let the study staff know and we will make sure that your infant's samples do not get stored for future research. We will not sell your infant's samples. Tests done on your infant's samples may lead to a new invention or discovery. We have no plans to share any money or other benefits resulting from this invention or discovery with you.

Please mark, initial and date one option:

_____I DO agree to have samples from my infant stored for future research
____________(signature/date)

_____I DO NOT agree to have samples from my infant stored for future research
_____________(signature/date)

____________________	________________________	______________
Witness Name (printed)	Witness Signature	Date

SUBJECT'S STATEMENT

This study has been explained to me. I volunteer to take part in this research, subject to the granting of relevant approvals. I have had a chance to ask questions. If I have questions later about the research, I can ask one of the researchers listed above. If I have questions about my rights as a research subject, I can call the Human Subjects Division at (206) 543-0098. I will receive a signed and dated copy of this consent form.

This study will not commence prior to obtaining the relevant local and international approvals.

____________________	________________________	______________
Mother's Name (printed)	Mother's Signature	Date
____________________	________________________	______________
Father's Name (printed)	Father's Signature	Date
____________________	________________________	______________
Name of Researcher obtaining consent (printed)	Signature of Researcher obtaining consent	Date
____________________	________________________	______________
Witness Name (printed)	Witness Signature	Date

Copies to: Parents, Researcher's file, Medical record (if applicable)
